# Identifying sepsis susceptibility genes in post-surgical patients using an artificial intelligence approach

**DOI:** 10.3389/fmed.2025.1644800

**Published:** 2025-12-15

**Authors:** Fernando Vaquerizo-Villar, Tamara Hernandez-Beeftink, María Heredia-Rodríguez, Esther Gómez-Sánchez, Mario Lorenzo-López, Rocío López-Herrero, Miguel Bardaji-Carrillo, Álvaro Tamayo-Velasco, Marta Martín-Fernández, Laura Sánchez-de-Prada, Julián Álvarez-Escudero, Sonia Veiras, Aurora Baluja, Hugo Gonzalo-Benito, Pedro Martínez-Paz, Adrián García-Concejo, Amanda Fernández-Rodríguez, María A. Jiménez-Sousa, Salvador Resino, Laura Martínez-Campelo, Eva Suárez-Pajés, Inés Quintela, Raquel Cruz, Ángel Carracedo, Jesús Villar, Carlos Flores, Roberto Hornero, Eduardo Tamayo

**Affiliations:** 1 Department of Anaesthesiology, Hospital Clínico Universitario de Valladolid, Valladolid, Spain; 2 Biomedical Engineering Group, University of Valladolid, Valladolid, Spain; 3 Centro de Investigación Biomédica en Red de Bioingeniería, Biomateriales y Nanomedicina (CIBER-BBN), Instituto de Salud Carlos III, Valladolid, Spain; 4 Division of Public Health and Epidemiology, School of Medical Sciences, University of Leicester, Leicester, United Kingdom; 5 BioCritic, Group for Biomedical Research in Critical Care Medicine, Valladolid, Spain; 6 Centro de Investigación Biomédica en Red de Enfermedades Infecciosas (CIBERINFEC), Instituto de Salud Carlos III, Madrid, Spain; 7 Department of Surgery, University of Valladolid, Valladolid, Spain; 8 Department of Haematology and Hemotherapy, Hospital Clínico Universitario de Valladolid, Valladolid, Spain; 9 Department of Cell Biology, Genetics, Histology and Pharmacology, Universidad de Valladolid, Valladolid, Spain; 10 Department of Anaesthesiology and Intensive Care Medicine, Clinical University Hospital of Santiago, Santiago de Compostela, Spain; 11 Sanitary Research Institute of Santiago (IDIS), Santiago de Compostela, Spain; 12 Servicio de Anestesiología, Hospital Virxe Da Xunqueira, A Coruña, Spain; 13 Research Support Unit, Hospital Clínico Universitario de Valladolid, Valladolid, Spain; 14 Centre for Experimental Medicine and Rheumatology, William Harvey Research Institute, Queen Mary University of London, London, United Kingdom; 15 Unidad de Infección Viral e Inmunidad, Centro Nacional de Microbiología (CNM), Instituto de Salud Carlos III, Majadahonda, Spain; 16 Centro de Investigación Biomédica en Red de Enfermedades Raras (CIBERER), Instituto de Salud Carlos III, Universidad de Santiago de Compostela, Santiago de Compostela, Spain; 17 Fundación Pública Galega de Medicina Xenómica, Servizo Galego de Saúde, Santiago de Compostela, Spain; 18 Research Unit, Hospital Universitario Nuestra Señora de Candelaria, Instituto de Investigación Sanitaria de Canarias (IISC), Santa Cruz de Tenerife, Spain; 19 CIBER de Enfermedades Respiratorias (CIBERES), Instituto de Salud Carlos III, Madrid, Spain; 20 Research Unit, Hospital Universitario Dr. Negrín, Las Palmas de Gran Canaria, Spain; 21 Li Ka Shing Knowledge Institute at St. Michael’s Hospital, Toronto, ON, Canada; 22 Faculty of Health Sciences, Universidad del Atlántico Medio, Tafira Baja, Las Palmas, Spain; 23 Genomics Division, Instituto Tecnológico y de Energías Renovables (ITER), Santa Cruz de Tenerife, Spain; 24 Facultad de Ciencias de la Salud, Universidad Fernando de Pessoa Canarias, Las Palmas de Gran Canaria, Spain

**Keywords:** explainable artificial intelligence (XAI), genome-wide association study (GWAS), sepsis, personalized medicine, surgical patients

## Abstract

**Background:**

Early detection of sepsis is essential for its successful management. Although genome-wide association studies (GWAS) have shown potential in identifying sepsis-related genetic variants, they often involve heterogeneous patient groups and use single-locus analysis methods. Here, we aim to identify new sepsis susceptibility loci in post-surgical patients using an explainable artificial intelligence (XAI) approach applied to GWAS data.

**Methods:**

GWAS was performed in 750 post-operative patients with sepsis and 3,500 population controls. We applied a novel XAI-based methodology to GWAS-derived single nucleotide polymorphisms (SNPs) to predict sepsis and prioritize new genetic variants associated with post-operative sepsis susceptibility. We also assessed functional and enrichment effects using empirical data from integrated software tools and datasets, with the top-ranked variants and associated genes.

**Results:**

Our XAI-GWAS approach showed a notable performance in predicting post-surgical sepsis and prioritized SNPs (such as rs17653532, rs1575081785, and rs74707084) with higher contribution to post-operative sepsis prediction. It also facilitated the discovery of post-operative sepsis risk loci with important functional implications related to gene expression regulation, DNA replication, cyclic nucleotide signaling, cell proliferation, and cardiac dysfunction.

**Conclusion:**

The combination of GWAS and XAI prioritized loci associated with post-operative sepsis susceptibility. The determination of key genes, such as *PRIM2*, *SYNPR*, and *RBSN*, through pre-operative blood tests could enhance risk stratification, enable early detection of post-operative sepsis, and guide targeted interventions to improve patient outcomes. Further research with additional and ethnically diverse cohorts comprising sepsis and non-sepsis patients undergoing major surgery is needed to validate these exploratory findings.

## 1 Introduction

Sepsis, a global health priority, is defined as a severe host response to a systemic infection, leading to a life-threatening organ dysfunction (1), with an incidence of approximately 189 adult cases per 100,000 population/annually (2, 3). The global mortality rate was around 17% in 2017, with significantly higher rates observed in patients with septic shock (4). Sepsis survivors may face significant functional and cognitive long-term disability (5) with important health and socioeconomic consequences. The use of sepsis bundles could improve survival (6, 7) although early identification of sepsis is mandatory, which is challenging even by experienced clinicians (6).

Multiple studies have focused on early detection of sepsis using information from patient demographics, vital signs, laboratory results, biosensors, and/or genetics (8–11). Since the host immune response to microbial agents is influenced by genetic variation (8), recent genome-wide association studies (GWAS) showed a potential to identify genomic variants associated with sepsis susceptibility (12), sepsis-associated acute respiratory distress syndrome (13), and sepsis-associated mortality (4, 14–17) in adults.

The genes identified in the few GWAS on sepsis patients differ among studies (4, 12–17), which could be mainly attributed to the heterogeneity of the patient populations involved. In this context, post-operative sepsis represents up to one-third of all sepsis patients (18) and has mortality rates that can reach up to 50% (3, 19). Its management involves high economic costs due to the need for mechanical ventilation and prolonged hospital stays (19, 20). Early diagnosis is complicated by the similarity of its symptoms to normal post-operative inflammatory responses (21), which delays timely intervention. Additionally, antibiotic resistance and the need for additional surgical interventions increase the complexity of treatment, especially in immunocompromised patients or those with multiple risk factors, where available biomarkers lack sufficient specificity for an accurate diagnosis (20). Postoperative sepsis is highly relevant for genetic studies, because the time of the precipitating insult is known (surgery). Hence, a preoperative assessment of genetic susceptibility to sepsis could enable risk stratification, guiding intensified monitoring or preventive interventions to better protect high-risk surgical patients (12, 22).

The existing GWAS of sepsis are characterized by the use of a single-locus analysis to select significant single-nucleotide polymorphisms (SNPs) (4, 12–17). In this respect, conventional methods may fail to capture complex interactions of SNPs with intermediate specificity that may have a higher contribution to the heritability of phenotypes (23, 24). Conversely, explainable artificial intelligence (XAI) approaches, which provide an ability to explain the decisions taken by complex artificial intelligence (AI)-based algorithms (25), have shown their usefulness to prioritize disease-associated genes (26–29). Specifically, these approaches have been previously applied for accurate phenotype prediction and susceptibility genes identification from GWAS-derived SNPs in complex diseases, such as atrial fibrillation (26), attention deficit hyperactivity disorder (27), hypertension, or diabetes (28).

In this exploratory study, we have analyzed genotype data from a large and homogeneous cohort of post-operative patients with sepsis and population controls to accurately predict sepsis and prioritize novel genetic variants associated with sepsis susceptibility. We hypothesized that XAI-based analysis of GWAS data could lead to the identification of new post-surgical sepsis susceptibility genes. Accordingly, our main objective was to obtain an XAI model that allows us to identify SNPs contributing to accurate prediction of post-operative sepsis and analyze their biological and functional implications, thereby facilitating their interpretation.

## 2 Materials and methods

### 2.1 Study design and participants

**Figure 1:**
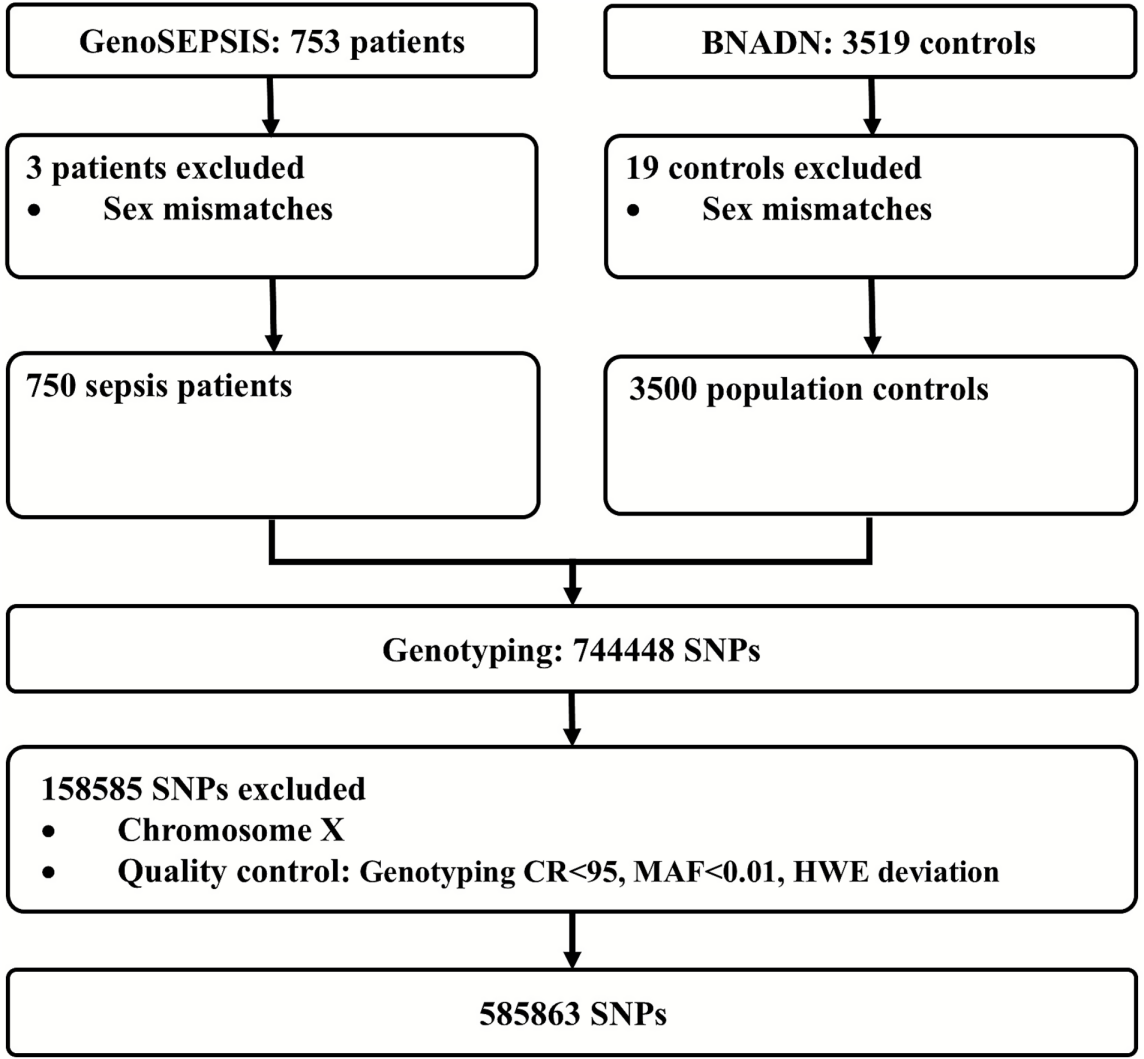
Study profile.

We performed a prospective cohort study using a sepsis patient cohort (GenoSEPSIS) and a population cohort from the Spanish National DNA Bank (BNADN) (Figure 1). The patient cohort GenoSEPSIS included 753 adult patients who underwent major surgery, admitted to two intensive care units (ICUs) in Spain from Hospital Clínico Universitario de Valladolid (HCUV) and Hospital Clínico Universitario de Santiago (CHUS) from November 2004 to December 2016. All patients were on mechanical ventilation and did not have any infection prior to surgery. Patients fulfilled the diagnosis of sepsis or septic shock according to SEPSIS-3 criteria (1) and the DNA extraction was performed within the first 24 h after the diagnosis of sepsis. Details of patient management and treatment, as well as data collection and follow-up, are included in the Supplementary methods and were also described for HCUV patients by Martín-Fernández et al. (30).

This study followed current Spanish legislation on biomedical research and the Declaration of Helsinki. Written informed consent was obtained from all participants or their representatives. The study was approved by the Ethics Committees for Clinical Research at participating centers (#No. PI 20–2070). The control cohort included genetic and demographic (sex and age) data available from 3,519 subjects from the BNADN, University of Salamanca, Spain^1^ and have been used in recent GWAS (31, 32). Subjects from the BNADN were unrelated individuals, uniformly distributed throughout different geographical areas of Spain, and lacking personal or family history of clinical conditions such as infectious diseases, cancer, circulatory disorders, endocrine issues, mental or behavioral disorders, as well as diseases affecting the nervous, visual, auditory, respiratory, and immune systems, among others. This cohort allows us to identify genetic variants that may be exclusively associated with sepsis predisposition, without the confounding influence of other comorbidities linked to critical illness.

### 2.2 SNP genotyping and preprocessing

A general scheme of the methods used in this study is presented in the Supplementary Figure S1. DNA samples from GenoSEPSIS and BNADN were genotyped at Centro Nacional de Genotipado-Universidad de Santiago de Compostela (CeGen-USC) using the Axiom Spain Biobank Array (Thermo Fisher Scientific). Genotyping quality control (QC) and filtering procedures are described in the Supplementary methods. A total of 585,863 SNPs from 750 sepsis patients and 3,500 population controls were obtained after QC analyses. Association analysis between SNP genotypes and sepsis was performed by PLINK 1.9, adjusting by age, sex, and the first two principal components (33) (Supplementary Figure S2). Different subsets of relevant SNPs were selected according to several thresholds of *p*-value (5 × 10^−2^, 5 × 10^−3^, 5 × 10^−4^, 5 × 10^−5^, 5 × 10^−6^, 5 × 10^−7^, and 5 × 10^−8^). See Supplementary methods for details.

### 2.3 XAI analysis and biological interpretation

XAI analysis was performed in two steps (Supplementary Figure S1). In the first step, a deep-learning model was designed to accurately predict sepsis from each subset of relevant SNPs. Specifically, a convolutional neural network (CNN) architecture, which has previously shown its usefulness in analyzing GWAS data (26, 27), was trained to automatically detect sepsis using previously subsets of selected SNPs (Supplementary Figure S3). To train the CNN, the whole dataset (4,250 samples) was randomly divided into training (50%), validation (25%), and test (25%) sets. The ratios of sepsis/control cases in these cohorts remained similar. See Supplementary methods for further details.

After obtaining the deep-learning model for sepsis prediction, the second step was the prioritization of sepsis-related SNPs. We applied the Deep SHAP XAI technique to obtain the SHAP values (34), which measure, for each patient, the contribution of each SNP to the prediction of sepsis. To find the most important SNPs contributing to sepsis, we took the average SHAP values (in absolute magnitude) from all patients accurately predicted as sepsis in the test set for each SNP. See Supplementary Figure S4 for further details.

For the top SNPs with the highest SHAP values for sepsis prediction, we assessed the functional *in silico* effects based on empirical data from different integrated software tools and datasets, and their association with the clinical characteristics of the sepsis patient cohort. Finally, we also performed a gene enrichment analysis querying different databases for the top SNPs and related genes. See Supplementary methods for further details.

### 2.4 Statistical analysis

The performance of the trained CNN models to detect sepsis (i.e., population control *vs.* sepsis) was assessed by the sensitivity (Se, proportion of sepsis subjects rightly classified), specificity (Sp, proportion of control subjects rightly classified), accuracy (Acc, proportion of subjects rightly classified), area under the receiver operating characteristic (ROC) curve (AUC), and odd ratio (OR). PLINK 1.9 was used to perform association analysis between SNPs and sepsis phenotype, as well as between the top SNPs identified by the proposed XAI methodology and various clinical characteristics (including comorbidities, diagnostic measurements, sources of infection, disease progression and hospital outcomes). For the gene enrichment analysis, the *z*-score of the deviation from the expected rank by the Fisher exact test was computed to assess statistically significant associations.

## 3 Results

### 3.1 Patient’s baseline characteristics

Table 1 shows the demographics of the sepsis patients and controls, where sepsis cases represented 17.6% (750 patients) with a median age of 72 (61–78) years and 65.9% of the proportion of males. Regarding sepsis patients, 83.9% (*n* = 629) had septic shock and their associated 90-day mortality rate was 42.7% (*n* = 320). Median SOFA and APACHE II scores were 9 (IQR 7–11) and 18 (IQR 15–22), respectively. A total of 561 patients (74.8%) had one or several associated comorbidities, including chronic cardiovascular disease (257 cases, 34.3%), chronic respiratory disease (156 cases, 20.8%), arterial hypertension (318 cases, 42.4%), chronic renal failure (89 cases, 11.8%), chronic liver failure (43 cases, 5.7%), diabetes mellitus (166 cases, 22.1%), obesity (109 cases, 14.5%), and immunosuppression (73 cases, 9.7%). Peritonitis (228 cases, 30.4%), pneumonia (185 cases, 24.7%), catheter (62 cases, 8.3%), and surgical wound (20 cases, 2.7%) are the main causes of infection.

**Table 1:** Baseline and clinical characteristics of patients with sepsis and population controls.

	Sepsis cohort (GenoSEPSIS)	Control cohort (BNADN)
Demographics		
Sex, males [*n* (%)]^a^	494 (65.9)	1,903 (54.4)
Age at sample intake [years, median (IQR)]^a^	72 (61–78)	47 (41–54)
Hospital site [*n* (%)]		
Number of subjects [*n* (%)]	750 (17.6)	3,500 (82.4)
HCUV	389 (51.9)	NA
CHUS	361 (48.1)	NA
Comorbidities [*n* (%)]		
Chronic cardiovascular disease	257 (34.3)	NA
Chronic respiratory disease	156 (20.7)	NA
High blood pressure	318 (42.4)	NA
Chronic renal failure	89 (11.9)	NA
Chronic hepatic failure	43 (5.7)	NA
Diabetes mellitus	166 (22.1)	NA
Obesity	109 (14.5)	NA
Immunosuppression	73 (9.7)	NA
Measurements at diagnosis median [IQR]		
Creatinine (mg/dl)	1.7 (1.1–3.1)	NA
White blood cells (cells/mm^3^)	12,685 (7,630–18,290)	NA
Lymphocytes (cells/mm^3^)	7.1 (4.5–11.6)	NA
Neutrophils (cells/mm^3^)	87.5 (81.6–91.3)	NA
SOFA score	9 (7–11)	NA
APACHE II score	18 (15–22)	NA
Source of infection [*n* (%)]		
Pneumonia	185 (24.7)	NA
Peritonitis	228 (30.4)	NA
Catheter	62 (8.3)	NA
Surgical site	20 (2.7)	NA
Others	245 (32.7)	NA
Time course and hospital outcomes		
Length of hospital stay [days, median (IQR)]	31 (19–44)	NA
Length of ICU stay [days, median (IQR)]	14 (7–21)	NA
Length of mechanical ventilation [days, median (IQR)]	9 (2–16)	NA
Severe sepsis [*n* (%)]	121 (16.1)	NA
Septic shock [*n* (%)]	629 (83.9)	NA
Mortality at 90 days [*n* (%)]	320 (42.7)	NA

^a^*p*-value < 0.05 between GenoSEPSIS and BNADN cohorts. IQR, interquartile range (25 percentile-75 percentile); SOFA, Sequential Organ Failure Assessment; APACHE II, Acute Physiology And Chronic Health Evaluation II; ICU, intensive care unit; HCUV, Hospital Clínico Universitario de Valladolid; CHUS, Hospital Clínico Universitario de Santiago; NA, Not applicable.

### 3.2 Identification of the most important SNPs for sepsis prediction

The sepsis prediction performance in the training, validation, and test sets of CNN models obtained with each subset of relevant SNPs are shown in Supplementary Tables S1–S3 and Supplementary Figure S5. Of note, the trained CNN model using SNPs with a *p*-value lower than 5×10^−3^ (3,761 SNPs) was considered the best model, achieving the highest accuracy in the validation (94.8%) set compared to CNN models derived using SNP subsets with *p*-values <5 × 10^−2^, 5 × 10^−4^, 5 × 10^−5^, 5 × 10^−6^, 5 × 10^−7^, and 5 × 10^−8^ (Supplementary Tables S1–S3). Notably, this model also achieved the highest accuracy on the test set, with an accuracy of 96.4%, an AUC of 0.985, a sensitivity of 85.6%, a specificity of 98.7%, and an odds ratio of 465.99.

**Figure 2:**
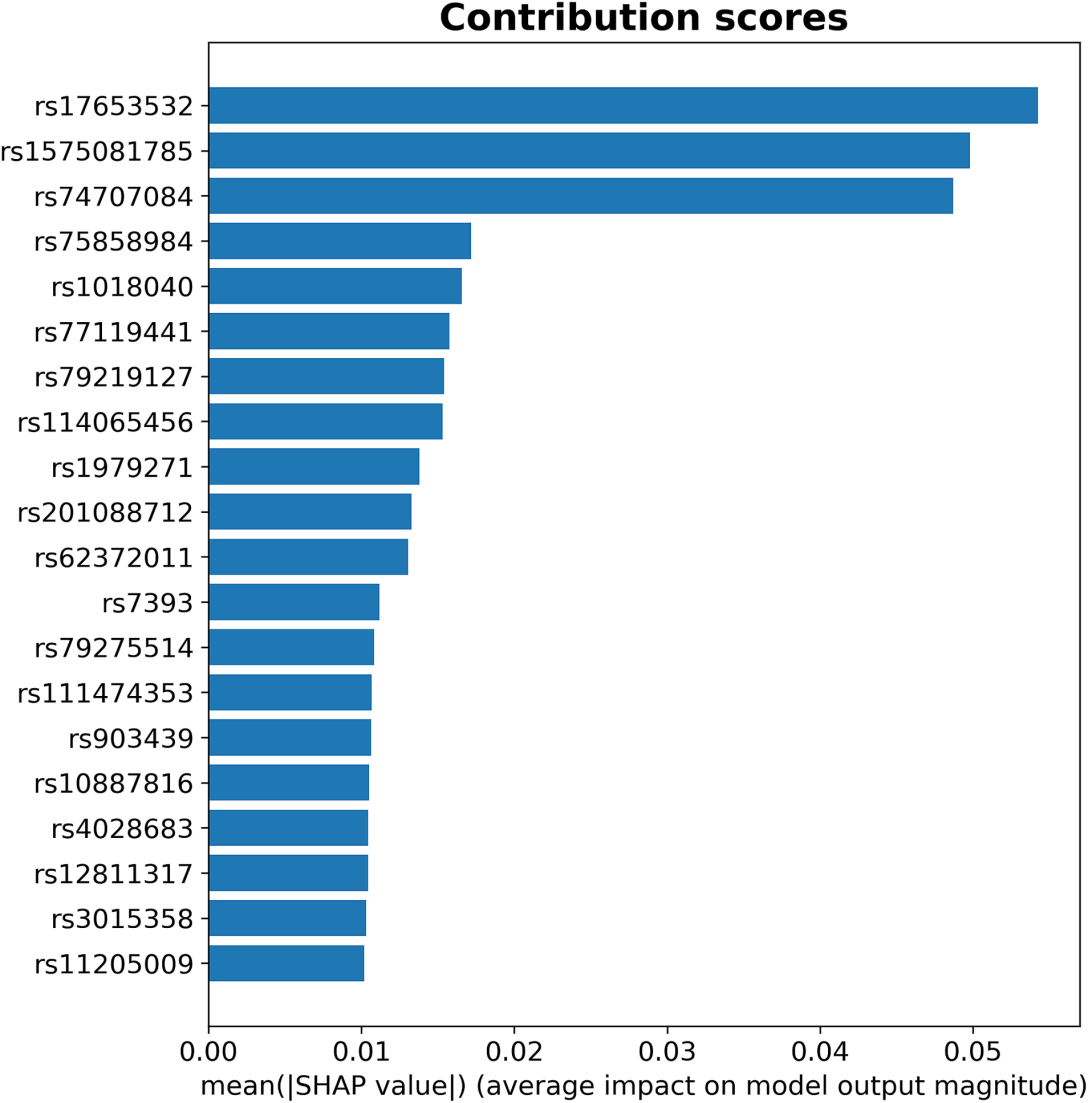
Contribution score of the top 20 SNPs to the prediction of sepsis.

Figure 2 shows the top 20 SNPs with the highest impact in the automatic prediction of sepsis in the whole test cohort, as determined using the contribution score (i.e., mean |SHAP value|). Notably, the sepsis prediction performance remains high using the top 20 SNPs (AUC = 0.951) or the top 3 SNPs (AUC = 0.886). The top 3 SNPs (rs17653532, rs1575081785, and rs74707084) had a high contribution to the detection of sepsis, with a considerably higher SHAP value (SHAP value >0.04) compared to the other top-ranked SNPs. Further details of the contribution score for each SNP, as well as sepsis prediction performance for different subsets of top SNPs in the test set are shown in the Supplementary Figure S6 and Supplementary Table S4.

### 3.3 *In silico* functional, clinical, and biological interpretation

We analyzed the *in silico* functional effect of the top 20 SNPs identified by the proposed XAI approach. Table 2 shows our findings from the 20 SNPs with the highest SHAP contribution value to the accurate prediction of sepsis. Among these variants, 11 were located within genes (two were missense, eight intronic, and one 3′ untranslated region) (Table 2). The intronic variant (rs17653532) with the highest SHAP contribution score for the accurate prediction of sepsis (SHAP value = 0.054) was located in the gene encoding the DNA Primase Subunit 2 (*PRIM2*). The second variant with the highest contribution (SHAP value = 0.050) was a missense variant (rs1575081785) within the gene encoding the Rabenosyn RAB Effector (*RBSN*). The third variant with a SHAP value of 0.049 (rs74707084) was an intronic SNP located within the gene encoding the Synaptoporin (*SYNPR*). Among the top 20 SNPs, and after Bonferroni correction for multiple-tests (*p*-value <0.0025), rs79219127, intronic to the *FAM155A* gene, showed statistically significant associations with the length of hospital (*p*-value = 2.6 × 10^−8^) and ICU stay (*p*-value = 7.7 × 10^−4^) in sepsis patients, whereas rs79275514, intronic to the gene encoding the Parkin protein (*PARK2*), was statistically significant related with high blood pressure (*p*-value = 2.1 × 10^−4^) and chronic hepatic failure (*p*-value = 3.2 × 10^−5^) comorbidities (Supplementary Table S6). We also found that most top-ranked 20 SNPs showed evidence of biological and regulatory effects on multiple elements related to chromatin state, changes in regulatory motifs, DNase I sensitivity, and expression quantitative trait loci (eQTLs) in different cell lines and tissues. More functional, clinical, and biological details are reported in the Supplementary Table S5.

**Table 2:** Top 20 SNPs with the highest SHAP contribution to the accurate prediction of sepsis.

Chromosome	Position (Hg37)	SNP	Located	Nearest genes	*p*-value^^^	SHAP values*
1	218705814	rs1018040	Downstream	*TGFB2/* *LYPLAL1*	1.69E-05	0.017
1	152453338	rs11205009	Upstream	*LCE5A*	2.87E-06	0.010
3	15115369	rs1575081785	Missense	*RBSN*	4.98E-40	0.050
3	63389846	rs74707084	Intron	*SYNPR*	1.28E-65	0.049
3	29607405	rs1979271	Intron	*RBMS3*	2.44E-03	0.014
5	59757497	rs62372011	Intron	*PDE4D*	2.28E-10	0.013
6	57189167	rs17653532	Intron	*PRIM2*	1.18E-03	0.054
6	162119549	rs79275514	Intron	*PARK2*	8.30E-04	0.011
6	30901543	rs111474353	Upstream	*DPCR1*	6.08E-59	0.011
6	166147479	rs903439	Intron	*PDE10A*	1.31E-05	0.011
7	150095271	rs7393	UTR-3	*ZNF775*	9.72E-04	0.011
10	90178820	rs10887816	Intron	*RNLS*	1.72E-04	0.011
11	121787956	rs77119441	Downstream	*SORL1*	1.47E-16	0.016
12	33598920	rs12811317	Upstream	*SYT10*	1.66E-20	0.010
13	108050599	rs79219127	Intron	*FAM155A*	4.09E-33	0.015
13	25480913	rs201088712	Missense	*CENPJ*	3.49E-29	0.013
13	105888343	rs3015358	Downstream	*DAOA-AS1*	1.78E-03	0.010
14	87312206	rs114065456	Downstream	*FLRT2/* *LINC01148*	4.96E-32	0.015
15	25037506	rs4028683	Downstream	*NPAP1*	4.36E-03	0.010
18	55016814	rs75858984	Upstream	*ST8SIA3*	1.09E-24	0.017

*Based on the contribution on sepsis. ^*p*-value resulted from the GWAS analysis. See information from 3,761 SNPs in the Supplementry Table S2 extended.

Based on the Gene Ontology annotation analysis, the most relevant and significantly enriched biological process was the negative regulation of heart contraction (*p*_adjusted_ = 0.02), involving two genes: Renalase, FAD Dependent Amine Oxidase (*RNLS*) and Phosphodiesterase 4D (*PDE4D*) (Supplementary Figure S7). We also found that the cyclic-nucleotide phosphodiesterase activity (*p*_adjusted_ = 1.55 × 10^−3^), the cyclic adenosine monophosphate (cAMP) binding (*p*_adjusted_ = 1.55 × 10^−3^), and the cyclic nucleotide binding (*p*_adjusted_ = 3.91 × 10^−3^) were the most significant enriched molecular process, which involved two genes (*PDE10A* and *PDE4D*) encoding phosphodiesterase proteins (Supplementary Figure S8). In terms of enrichment in the Jessen disease database, acrodysostosis (*p*_adjusted_ = 0.068) and dementia (*p*_adjusted_ = 0.068) were the most relevant diseases, involving *PDE4D*, Sortilin Related Receptor 1 (*SORL1*) and Parkin RBR E3 Ubiquitin Protein Ligase (*PARK2*) genes (Supplementary Figure S9). In the expression heatmap across different tissues, we observed that *RBSN*, Lysophospholipase Like 1 (*LYPLAL1*), and Zinc Finger Protein 775 (*ZNF775*) genes were highly expressed in almost all tissues (Supplementary Figure S10). More details are reported in the Supplementary results.

## 4 Discussion

To the best of our knowledge, this is the first XAI approach applied to GWAS-derived SNPs for patients with post-operative sepsis. We identified top-ranked SNPs with higher contribution to sepsis prediction involved in chromatin regulation, regulatory motifs, DNase I sensitivity, and significant eQTLs in different cell lines and tissues (including blood, fibroblasts, and immune response cells). Among the top20-ranked SNPs, we found SNPs associated with inflammation, blood cell count, and sepsis traits, and in sensitivity analysis of the sepsis cohort, we observed SNPs associated with hospital and ICU stay length, chronic liver failure, and hypertension. This exploratory study allowed the prioritization of three potential biomarkers with the highest contribution to post-surgical sepsis prediction (rs17653532, rs1575081785, and rs74707084) located in *PRIM2, RBSN,* and *SYNPR* genes, which are involved in integrative processes, such as gene expression regulation, DNA replication, and cell proliferation.

The SNP with the highest SHAP contribution was an intronic variant located in the *PRIM2* gene involved in DNA replication (35), which is critical in sepsis for its role in apoptosis, oxidative stress, and metabolic changes. *PRIM2* has been related to consecutive trauma-induced sepsis based on an expression profiling analysis (36). The second SNP with the highest SHAP contribution score is in the *RBSN* gene, which encodes a protein from the FYVE zinc finger family and is involved in vesicle trafficking. Zinc finger proteins may contribute to inflammation, immune cell function, and tissue repair by modulating gene expression and regulating key immune-related genes (37–39). In the context of sepsis, these proteins may also affect cellular dysfunction, apoptosis, and DNA repair mechanisms, thereby affecting cell survival and tissue integrity (39). Similarly, a gene co-expression network analysis identified a zinc finger family gene (*ZNF721*) in a gene cluster for septic shock patients (40). Finally, a third gene, *SYNPR,* which encodes Synaptoporin, a protein found in the central nervous system, is involved in synaptic vesicle trafficking and neurotransmitter release (41).

In addition, our analyses revealed two genes encoding phosphodiesterases (*PDE10A* and *PDE4D*), involved in cyclic nucleotide signaling (42, 43). Cyclic nucleotide signaling is involved in several cellular processes, including immune response and inflammation (44), and phosphodiesterase inhibitors have shown potential therapeutic effects in experimental models of sepsis and lung inflammation (45, 46). In fact, cyclic-nucleotide phosphodiesterase activity and the cAMP binding were the most enriched relevant molecular processes in our study. Furthermore, the most significantly enriched biological process was negative regulation of heart contraction, involving *RNLS* and *PDE4D* genes. Cardiac dysfunction is an important consequence of sepsis, caused by increased inflammation or suppression of fatty acid and glucose oxidation, or due to adenosine triphosphate (ATP) depletion (47, 48). Although we did not find any previously significant associated variants with sepsis in our study, some of the ranked SNPs (rs201088712, rs3015358, and rs114065456) were associated with white blood cell count, sepsis, and sepsis-associated death in the UK Biobank. Thus, we have identified new possible candidate genes associated with post-operative sepsis susceptibility, highlighting the role of genes related to gene expression, DNA replication, cyclic nucleotide signaling, cell proliferation, and cardiac dysfunction.

Comprehensive XAI approaches have identified clinical features (vital signs, laboratory values, or demographics, among others) from electronic health records (EHR) contributing to early detection of sepsis (49–51) but they have not been applied to sepsis-related genomic data until now. Several GWAS studies have identified SNPs and evaluated polygenic risk scores (PRS) associated with sepsis susceptibility and mortality (4, 12–17), offering insights that could inform early prevention and treatment strategies targeting sepsis-related complications. However, only Engoren et al. (12) described a polygenic risk score for sepsis susceptibility, achieving an AUC of 0.752. This sepsis prediction performance in Engoren et al. (12) could be attributed to: (i) including both sepsis-2 and sepsis-3 adult perioperative patients identified from EHR data; (ii) using peri-operative controls identified from a university EHR, who may present confounding comorbidities; (iii) applying a different sepsis prediction model than in our study (logistic regression *vs*. CNN), which may not capture complex SNP interactions contributing to sepsis predisposition. Regarding the methodology in existing GWAS for sepsis (4, 12–17), it is noteworthy that the SNPs most strongly associated with the phenotype (i.e., those with the lowest *p*-values) could not contribute to the most predictive genetic signature. Instead, the most predictive signature often consists of SNPs that provide complementary information (23). In this respect, our XAI methodology relying on a *p*-value <5 × 10^−3^ threshold prioritized those SNPs that have a higher influence for phenotype prediction. Among the top 20-ranked SNPs, we found both SNPs with high (i.e., *p*-value from GWAS <5 × 10^−8^) and intermediate specificity (i.e., *p*-value >5 × 10^−8^), being one of the SNPs with intermediate specificity also in the top 3 SNPs (rs17653532 (*PRIM2*), GWAS *p*-value = 1.18 × 10^−3^), thus confirming the limitations of using the standard threshold of 5 × 10^−8^ for statistical significance in GWAS studies (23). Importantly, our XAI approach prioritized three SNPs (rs17653532, rs1575081785, and rs74707084) with a high contribution to sepsis prediction (i.e., highest SHAP values) and good performance when validated in an independent test subset (AUC = 0.886), highlighting the potential role of these variants and related genes in sepsis risk in post-surgical patients. In clinical practice, the output probability threshold of the sepsis prediction models could be adjusted to identify patients at higher or lower risk of sepsis, prioritizing either sensitivity (to capture more high-risk patients) or specificity (to reduce false positives), depending on the intended clinical application and available resources (52). Our results agree with previous XAI approaches applied to analyze GWAS-derived SNPs in complex diseases, such as atrial fibrillation (26), attention deficit hyperactivity disorder (27), hypertension, or diabetes (28).

Although the *in silico* functional findings suggest potential implications for early and personalized prevention and treatment for post-operative sepsis, further biological validation of these exploratory findings through *in vitro* and *in vivo* analyses is required for clinical relevance and generalizability. XAI provides a powerful approach to prioritize SNPs for sepsis prediction, providing insights into cohort-wide and individual-specific genetic predispositions. We also highlighted the clinical and biological relevance of genes associated with the top 3 SNPs (*PRIM2*, *RBSN,* and *SYNPR*). Identifying these genetic variants in a preoperative blood test could help in the early detection of sepsis in surgical patients, may enable risk stratification, and allow prompt pharmacological treatment, thus reducing mortality and long-term disability. This concept is in line with the recent clinical study by Liesenfeld et al. (53), who clinically validated an AI-driven blood test using host mRNAs expression data to predict acute infection and sepsis. However, our findings remain exploratory and further external clinical validation is crucial to confirm their reproducibility and generalizability of our findings. Studies in ethnically diverse cohorts are also warranted, given that most existing sepsis GWAS have been conducted in populations of European ancestry and genetic associations often fail to replicate across ancestral group (54, 55).

We acknowledge some strengths and limitations of our study. The existing GWAS in sepsis patients include patients with sepsis of any etiology (4, 12–17), which leads to the identification of genes that differ among studies (4, 12–17). In contrast, we focus exclusively on cases based on patients with postsurgical sepsis, making our population highly homogeneous and with applicable results within this context. Nevertheless, patient heterogeneity persists due to differences in surgical procedures, infection sources, or host responses (56–58). Thus, future studies are warranted to investigate whether specific post-operative patient subgroups exhibit distinct genetic associations or clinical outcomes. Apart from this, the study samples were collected from two hospitals, and the sample size was not very large due to the challenging nature of data collection, and rare variants in or near identified regions may go undetected due to technological limitations. In this respect, the use of a test set derived from the same underlying cohort represents a potential limitation regarding external generalizability. However, the adoption of a hold-out validation scheme (with independent training, validation, and test subsets) provides a robust internal assessment of model performance in the absence of an available external or independent postsurgical sepsis cohort, that reduces the risk of overfitting. This validation approach has been shown to yield generalizable, reproducible and biologically meaningful results in recent AI-based genomic studies (26–28). Nevertheless, the validation in a large, independent, geographically, and ancestrally distinct dataset of post-operative patients with diverse ancestries would be a necessary future step to confirm our findings. This would also allow consideration of additional factors such as comorbidities, measurements at diagnosis, and sources of infection in sepsis prediction. Genome and whole exome sequencing analyses would also provide a better resolution to achieve this goal. Regarding the proposed XAI methodology, we used a CNN for sepsis prediction, originally designed for image analysis. Recent studies have shown that CNNs are suitable to analyze GWAS data (26, 27). Another limitation is the use of population controls who were not clinically evaluated for sepsis. As such, we cannot entirely exclude the possibility that some controls could have been cases exposed to relevant environment factors or survivors of a previous sepsis episode. However, the use of population controls provided access to a well-characterized and substantial sample size cohort, thereby increasing the statistical power of the study, and they are commonly utilized in large-scale GWAS studies of infectious diseases (31, 32, 59–61). Notably, the COVID-19 Host Genetics Initiative consortium studies demonstrated that genetic analyses using various comparisons of controls with COVID-19 severity (e.g., infection, hospitalization, critical illness) yield overlapping results, supporting the validity of using population controls in genetic research on infectious diseases (59). Similarly, several GWAS of infectious conditions have successfully relied on population-based biobanks as control sources when disease-specific non-affected cohorts were unavailable, highlighting the practical and methodological acceptance of this approach in complex traits (31, 32, 60, 61). Conversely, although this has been observed in other studies, and we adjusted for sex and age in the association analysis to reduce bias due to age imbalance between cases and controls, young patients may eventually undergo surgery and develop sepsis. Moreover, the genotype by environment interactions may show challenges in this kind of study due to the dynamic influence of the environment on gene expression. As a result, the effects of genetic variants could be masked or modified by environmental factors. Thus, future studies should include clinical validation in postsurgical or critically ill patients without sepsis as controls once reliable genetic data is available, to better delineate genetic variants specifically associated with sepsis susceptibility within the context of critical illness.

In conclusion, our XAI approach applied to GWAS-derived SNPs enabled the identification of significant risk loci associated with post-surgical sepsis that could be implemented in clinical practice for improving patient outcomes. We found variants with functional, regulatory and clinical implications, as well as genes related to gene expression regulation, DNA replication, cyclic nucleotide signaling and cell proliferation, and cardiac dysfunction, among other biological processes. We also identified three potential biomarkers with the highest contribution to sepsis prediction (rs17653532, rs1575081785, and rs74707084), located in *PRIM2*, *RBSN* and *SYNPR* genes, which could be determined in a preoperative blood test, allowing targeted and precise interventions to prevent and treat sepsis in patients undergoing surgery. Further investigations, including *in vitro* and *in vivo* analyses, as well as complementary studies in cohorts comprising sepsis and non-sepsis patients undergoing major surgery will be needed to optimally evaluate the genetic factors contributing to sepsis predisposition and to provide an external validation of our exploratory findings.

## Data Availability

The data analyzed in this study is subject to the following licenses/restrictions: GWAS are not publicly available, but it can be obtained upon reasonable request from the authors. Requests to access these datasets should be directed to Eduardo Tamayo, eduardo.tamayo@uva.es.

## Footnotes

1
http://www.bancoadn.org
