# Supplementary material for: Identifying sepsis susceptibility genes in post-surgical patients using an artificial intelligence approach
Source: Front Med (Lausanne). 2025 Dec 15;12:1644800. doi: 10.3389/fmed.2025.1644800 (PMC12745203; doi:10.3389/fmed.2025.1644800)
Supplement: Supplementary file 4 [file Data_Sheet_1.DOCX]

Supplementary Material

[**1 Supplemental Methods 2**](#_Toc200466166)

[**1.1 Study population and samples 2**](#_Toc200466167)

[**1.1.1 GenoSEPSIS population 2**](#_Toc200466168)

[**1.1.2 BNADN population 3**](#_Toc200466169)

[**1.2 Genotyping, quality control and association analysis 3**](#_Toc200466170)

[**1.3 XAI analysis 4**](#_Toc200466171)

[**1.3.1 Sepsis prediction 4**](#_Toc200466172)

[**1.3.2 Identification of sepsis-related SNPs 5**](#_Toc200466173)

[**1.4 Functional, clinical, and biological interpretation 6**](#_Toc200466174)

[**2 Supplemental Results 7**](#_Toc200466175)

[**2.1 Identification of the most important SNPs for sepsis prediction 7**](#_Toc200466176)

[**2.1.1 Sepsis prediction performance for different subsets of relevant SNPs 7**](#_Toc200466177)

[**2.1.2 Contribution score of each SNP for sepsis detection 7**](#_Toc200466178)

[**2.1.3 Sepsis prediction performance for different subsets of top SNPs 7**](#_Toc200466179)

[**2.2 *In silico* functional, clinical, and biological interpretation 8**](#_Toc200466180)

[**2.2.1 Functional analysis 8**](#_Toc200466181)

[**2.2.2 Clinical implications 9**](#_Toc200466182)

[**2.2.3 Enrichment analysis 9**](#_Toc200466183)

[**3 Supplemental Tables 11**](#_Toc200466184)

[**4 Supplemental Figures 19**](#_Toc200466185)

[**5 Supplemental References 29**](#_Toc200466186)

# Supplemental Methods

**Figure S1** shows a general scheme of the proposed methodology.

## Study population and samples

### GenoSEPSIS population

GenoSEPSIS is a prospective cohort of 753 adult patients who underwent major surgery and were admitted to two intensive care units (ICUs) in Spain, one at the Hospital Clínico Universitario de Valladolid (HCUV) and at the Hospital Clínico Universitario de Santiago (CHUS) from November 2004 to December 2016. All patients were on mechanical ventilation and did not have any infection prior to surgery. Sepsis or septic shock diagnosis was clinically defined according to SEPSIS-3 definitions (1). Before enrollment, written informed consent was obtained from all participants or their representatives, and the Ethics Committee for Clinical Research at the Hospital Clínico Universitario de Valladolid, Spain and the Ethics Committee for Clinical Research at the Hospital Clínico Universitario de Santiago, Spain approved the study (#No. PI 20-2070). This study adhered to the current Spanish legislation on biomedical research and The Code of Ethics of the World Medical Association (Declaration of Helsinki).

Patients were subjected to management and treatment in accordance with prevailing guidelines for general critical care (1), which include the following: (i) early identification of the causative microorganism, with optimization of intravenous antibiotic selection and timely administration based on antibiogram findings; (ii) individualized fluid resuscitation and vasopressor utilization, with the specific objective of maintaining a systolic blood pressure of ≥90 mmHg or a mean arterial pressure of ≥65 mmHg; (iii) maintenance of hemoglobin levels between 7 and 10 g/dL, taking into consideration the patient's overall clinical condition (2). The choice of drugs for sedation and analgesia, hemodynamic management modalities, and the decision to perform tracheostomy were left to the discretion of the attending physician. Weaning from mechanical ventilation was initiated when the attending physician considered it clinically appropriate. Additionally, routine gastric protection was provided during the initial 24 h of ICU stay through the administration of omeprazole (20 mg/iv). More details are described previously by Martín-Fernández et al. (3), who used clinical data from the HCUV to examine whether PaO_2_ is associated with risk of death in adult patients with sepsis/septic shock after major surgery.

Throughout the study period, patients admitted to participating in ICUs underwent daily screening to identify eligible candidates and determine the onset of severe sepsis/septic shock. A specifically standardized form was employed to systematically gather demographic and clinical data, encompassing DNA samples and hematological, biochemical, radiological, microbiological, and biomarker measurements within the initial 24 h following sepsis/septic shock diagnosis. Assessment of illness severity was assessed using the Sequential Organ Failure Assessment (SOFA) scale (4) and the Acute Physiology and Chronic Health Evaluation II (APACHE II) (5) score. The diagnostic criteria for sepsis and septic shock adhered to the guidelines presented in the 3rd International Consensus Definitions for Sepsis and Septic Shock (Sepsis-3) (1). Sepsis was defined as a life-threatening organ dysfunction stemming from a dysregulated host response to infection, wherein organ dysfunction was manifested by a rise in the SOFA score of ≥2 points. Septic shock, a subgroup of sepsis, was characterized by the requirement for vasopressors to maintain a mean arterial pressure of ≥ 65 mmHg and a serum lactate level >2 mmol/L (>18 mg/dL) in the absence of hypovolemia. More details on the diagnosis and definitions of patients recruited for the study are well described by Martín-Fernández et al. (3).

### BNADN population

The control cohort included genetic and demographic (sex and age) data available from 3,519 subjects from the BNADN, University of Salamanca, Spain (<http://www.bancoadn.org>) and have been used in recent largest related severe or critical pneumonia or equivalent GWAS (6,7). Subjects from the BNADN were unrelated healthy individuals, uniformly distributed throughout different geographical areas of Spain, and lacking personal or family history of clinical conditions such as infectious diseases, cancer, circulatory disorders, endocrine issues, mental or behavioral disorders, as well as diseases affecting the nervous, visual, auditory, respiratory, and immune systems, among others.

## Genotyping, quality control and association analysis

Genomic DNA was obtained from peripheral blood isolated using the Chemagic DNA Blood 100 kit (PerkinElmer Chemagen Technologies GmbH), following the manufacturer’s recommendations. Samples were genotyped with the Axiom Spain Biobank Array (Thermo Fisher Scientific) following the manufacturer’s instructions in the Santiago de Compostela Node of the National Genotyping Center (CeGen-ISCIII; http://www.usc.es/cegen). This array contains around 781,759 probes for genotyping a total of 756,834 SNPs. Clustering and genotype calling were performed jointly for both cohorts (753 sepsis patients from GenoSEPSIS and 3,519 population controls from BNADN) using the Axiom Analysis Suite v4.0.3.3 software.

A quality control (QC) procedure using PLINK 1.9 was applied to both samples and genotyped SNPs. Samples from subjects with sex mismatches were first excluded. Variants with minor allele frequency (MAF) <1%, call rate <98%, and markers significantly deviating from Hardy-Weinberg equilibrium expectations (p<10^-6^) with mid-p adjustment were excluded. In addition, excess heterozygosity was assessed to eliminate potential cross-sample contamination, and samples missing >2% of variants were filtered out. Subsequently, autosomal SNPs were retained, and regions of high linkage disequilibrium (LD) were removed using LD pruning (1,000 SNPs per window, step size of 80, and r2 threshold of 0.1) to assess relationships and estimate global ancestry proportions. Kinship was assessed using identity by descent (IBD) scores to remove each pair with PI_HAT>0.25 showing a Z0, Z1, and Z2 coherent pattern (according to theoretical expected values for each level of relatedness). Genetic principal components (PCs) were calculated using PLINK with the subset of LD-pruned variants.

A total of 585,863 SNPs from 750 sepsis patients and 3,500 population controls were obtained after QC analyses. Association analyses were performed using PLINK 1.9 (8), adjusting for age, sex, and the two main principal components (**Figure S2**). In this way, different subsets of relevant SNPs were selected according to different *p*-value thresholds: 5x10^-2^ (33,596 SNPs), 5x10^-3^ (3,761 SNPs), 5x10^-4^ (495 SNPs), 5x10^-5^ (100 SNPs), 5x10^-6^ (37 SNPs), 5x10^-7^ (30 SNPs), 5x10^-8^ (28 SNPs).

## XAI analysis

### Sepsis prediction

The first step of the XAI methodology consists of the application of a deep-learning methodology to predict sepsis from GWAS data. In the last decade, deep-learning algorithms have emerged as a powerful tool to improve the predictive performance in many fields (9), with some recent studies predicting complex diseases from GWAS data (10–12). In this study, we build a convolutional neural network (CNN) model to automatically identify sepsis from each subset of relevant SNPs.

**Figure S3** shows the overall CNN-based architecture employed in this study. Based on their genotype, the input SNPs data are first transformed into a numerical format using a one-hot representation (**Figure S3A**), so each SNP is transformed into a numerical 1x4 vector: AA to 1000, Aa or aA to 0100, aa to 0010 and NA (missing) to 0001, where we assume A is the major allele, and a is the minor allele. Thus, for each sample, the input subset of relevant SNPs (L SNPs) is transformed into a Lx4 matrix.

This Lx4 encoded SNP input is then processed using five stacked convolutional blocks (conv block), each one composed of the following layers (**Figure S3B**): a 1-D convolution, batch normalization (BN), activation, and dropout (13). In the first conv block, the 1-D convolution (1-D conv) layer extracts intra-SNP features using 64 filters of size 1x4. Conversely, the 1-D conv layer of the four remaining conv blocks are intended to learn inter-SNP features using 64 filters of size 4x1 and a stride of 4, which reduces dimensionality. In each block, a batch normalization (BN) layer follows the 1-D convolution to normalize the extracted features. Then, a Rectified Linear Unit (ReLU) activation function was used to introduce nonlinearity to the normalized feature maps. Finally, a dropout layer is included in each convolutional block to prevent overfitting by randomly removing network connections with a probability of $p_{drop}$ (13). The value of $p_{drop}$ was empirically determined in each model as the one that maximized the performance of the CNN in the validation set.

After the last convolutional block, the output block consists of a flattening layer, which reshapes the 2D feature maps into 1D data, and a softmax activation function applied to obtain the probability of belonging to sepsis and control groups for the input SNP data.

Prior to train the CNN, the whole dataset (4,250 samples) was randomly divided into three sets: training (50%), used to train the CNN models for sepsis prediction, validation set (25%), used to monitor the convergence of the models and hyperparameter selection, and test set (25%), used to evaluate the performance. Accordingly, our CNN models for sepsis prediction are validated into a subset of patients that were not used to train and optimize the AI-based methodology, thus reducing model bias. The CNN architecture was trained on a NVIDIA GeForce RTX 2080 GPU with the following configuration (13): He-normal method for network weights initialization; Adam algorithm with an initial learning rate of 0.001 for the optimization of network weights; binary cross entropy as the loss function to minimize; batch size of 128 with a random data shuffling strategy to feed training data in random order into the GPU memory; reduction of the learning rate by a factor of 2 after 10 epochs of non-improvement in the validation loss; and early stopping after 40 epochs of non-improvement, restoring the model to the best weights in the validation set.

The performance of the trained CNN models was assessed by the sensitivity (Se, proportion of sepsis subjects rightly classified), specificity (Sp, proportion of control subjects rightly classified), accuracy (Acc, proportion of subjects rightly classified), and area under the receiver operating characteristic (ROC) curve (AUC).

### Identification of sepsis-related SNPs

After obtaining the CNN-based deep-learning model for sepsis prediction, the second stage of XAI analysis involves interpreting the CNN to identify those SNPs that have a higher influence in the prediction of sepsis. In the last few years, XAI techniques have gained increasing attention due to their capability to explain artificial intelligence (AI)-based models (including deep-learning ones) *a posteriori* (14), with some recent studies applying them to explain deep-learning model for diseases prediction and subsequently identifying potential risk genes for disease susceptibility from GWAS data (10–12). In this study, we apply the Deep SHAP explanation approach to interpret the CNN-based deep-learning model and obtain those SNPs with the highest contribution to the prediction of sepsis (15).

Deep SHAP is included within the ‘SHapley Additive exPlanations’ (SHAP) approach. This unified framework relies on assigning importance values (termed SHAP values) to the features obtained and is model agnostic, which means that it does not depend on the deep-learning method used. One of the most interesting things of SHAP is that it brings together previous methods (e.g., LIME, DeepLIFT, Shapley values, or Kernel SHAP) that have shown to be helpful in the interpretability of models trained with biomedical data (15).

Deep SHAP is a specific technique for deep-learning models that relies on the combination of Deep LIFT and Shapley values in which the latter are not exactly obtained but only estimated (15). The role of Deep LIFT is back-propagating to each input feature the contribution for a particular output of each neuron, thus assigning them an importance score that is ultimately associated to a SHAP value. Deep SHAP has been recently used in a GWAS to find risk genes associated with attention deficit hyperactivity disorder (12), as well as to obtain the most important genes for cancer classification using high-dimensional omics data (16). The authors in these studies showed great potential for novel biomedical knowledge discovery from deep-learning models (12,16).

**Figure S4** presents a scheme of the proposed Deep SHAP approach for identifying sepsis-related SNPs. As for the implementation, the trained CNN model, together with the training data acting as background, are passed to DEEP SHAP. By integrating over numerous background samples, DEEP SHAP provides an explainer object that calculates an accurate estimation of SHAP values. The sum of these approximate SHAP values equals the differences between the expected model output based on the provided background samples and the current model output (15).

Then, the obtained DEEP SHAP explainer object is applied to the test data. For each sample in the test set, a SHAP values matrix with the same dimension as the input can be calculated using Deep SHAP explainer object. Given a test sample of N subjects, each with a Lx4 encoded SNP input (L SNPs encoded to a 1×4 vector), and by utilizing the DEEP SHAP explainer object, a SHAP values matrix of dimensions NxLx4 is generated.

Based on this SHAP value matrix, it is possible to identify for each patient the SNPs with a higher contribution to the detection of sepsis by taking the average of its four SHAP values (NxL matrix). Furthermore, to obtain the overall contribution of each SNP for sepsis prediction in the whole cohort, the absolute values of SHAP values were averaged over all samples accurately predicted as sepsis in the test set for each SNP, resulting in a SHAP values vector of Lx1 dimension.

## Functional, clinical, and biological interpretation

We conducted a comprehensive assessment of functional effects using empirical data obtained from various integrated software tools and datasets. To determine the regulatory potential and rank the functional roles of each SNP and its associated gene, we used HaploReg v4.1 (17), RegulomeDB (18), and The Open Targets Post-GWAS portal (19). For local expression quantitative trait loci (eQTLs), we leveraged the Genotype-Tissue Expression Project (GTEx) (20). Furthermore, we used PhenoScanner (<http://www.phenoscanner.medschl.cam.ac.uk/>) facilitated the cross-referencing of genetic variants with a broad range of phenotypes.

To assess clinical implications, we have analyzed the association of the most relevant SNPs with clinical characteristics of the patients with sepsis in the GenoSEPSIS cohort. Accordingly, association analysis between the top 20 SNPs with the highest SHAP value for sepsis prediction and clinical characteristics (comorbidities, measurements at diagnosis, sources of infection, and time course and hospital outcomes) was performed by PLINK 1.9, adjusting by age, sex, hospital site, and the first two principal components. For each clinical variable, association analysis was only performed using the subjects with available information (i.e., non-missing value). Bonferroni correction was used to obtain adjusted *p*-values. Thus, instead of the traditional *p*-value<0.05, the required significance level was obtained by dividing it by the number of comparisons. Since the number of tests was 20 (i.e., the top 20 SNPs), the Bonferroni-adjusted significance level was 0.05/20 = 0.0025.

Finally, we conducted an enrichment analysis using the Enrichr tool (<https://maayanlab.cloud/Enrichr/>), querying Jensen Disease, Gene Ontology Molecular Function (2023), and Gene Ontology Biological Process (2023) databases (21). We also employed Functional Mapping and Annotation of Genome-Wide Association Studies (FUMA) to annotate the functional mapping and expression patterns of each gene across diverse cell types (22,23).

# Supplemental Results

## Identification of the most important SNPs for sepsis prediction

### Sepsis prediction performance for different subsets of relevant SNPs

**Table S1, Table S2, and Table S3** show the sepsis prediction performance of the CNN models obtained with each subset of relevant SNPs in the training, validation, and test sets, respectively. It can be observed that the performance of the CNN models is slightly higher in the training set than in the validation and test sets, and these differences tend to increase as the number of input SNPs grows, reflecting the high-dimensionality problem. Interestingly, Liu et al., (12) and Mieth et al., (11), who applied AI approaches to identify the most important SNPs for phenotype prediction, also reported 100% training accuracy. This is a common issue in deep-learning models, known as overfitting (13), which we tried to minimize by using early stopping and dropout strategies. Conversely, the sepsis prediction performance is similar in the validation and test sets, which underscores the generalizability of the CNN models to independent subsets of patients, agreeing with the findings reported by Liu et al., (12) and Mieth et al., (11). Notably, the CNN model trained using SNPs with a *p*-value lower than 5x10^-3^ (3,761 SNPs) was considered the best model, as it achieved the highest accuracy in the validation (94.8%) set compared to CNN models derived using SNP subsets with *p*-values < 5x10^-2^, 5x10^-4^, 5x10^-5^, 5x10^-6^, 5x10^-7^, and 5x10^-8^ (**Table S2**). Specifically, this CNN model also reached the highest performance in the test set, with an accuracy of 96.4%, an AUC of 0.985, a sensitivity of 85.6%, a specificity of 98.7%, and an odds ratio of 465.99 (**Table S3** and **Figure S5**). Accordingly, the Deep SHAP XAI method was applied to this CNN model to assess the contribution of each SNP to the prediction of sepsis.

### Contribution score of each SNP for sepsis detection

**Table 2 extended,** included as a supplementary excel file, shows the contribution score (i.e., mean |SHAP value|) for each SNP to the prediction of sepsis. In contrast, **Figure S6** shows the log-scale histogram of the SNP contribution scores in the test set. Interestingly, a power-law distribution was observed in the contribution scores, suggesting that most of the input features (SNPs) had little relevance in the prediction of sepsis.

### Sepsis prediction performance for different subsets of top SNPs

**Table S4** shows the sepsis prediction performance in the test set for different subsets of top SNPs. As expected, sepsis prediction performance increases with the number of top SNP selected. Nonetheless, a remarkable performance is also obtained using subsets with a reduced number of top SNPs. For instance, the sepsis prediction performance remains high using the top 20 SNPs (AUC=0.951) or the top 3 SNPs (AUC=0.886). Looking at **Table S3** and **Table S4**, it can be seen that sepsis prediction performance is higher with the top 20 SNPs (Accuracy=94.5%, AUC=0.951) than SNP subsets with *p*-values < 5x10^-6^ (37 SNPs, Accuracy=93.4%, AUC=0.937), 5x10^-7^ (30 SNPs, Accuracy=93.4%, AUC=0.928), and 5x10^-8^ (28 SNPs, Accuracy=92.8%, AUC=0.913). Furthermore, sepsis prediction performance using the top 20 SNPs (Accuracy=94.5%, AUC=0.951) is similar to the obtained with SNP subsets with *p*-values < 5x10^-4^ (495 SNPs, Accuracy=94.0%, AUC=0.962), 5x10^-5^ (100 SNPs, Accuracy=94.1%, AUC=0.955), which have a considerably higher number of SNPs that do not improve sepsis prediction. This highlights the usefulness of the proposed XAI-GWAS methodology to prioritize SNPs with a higher contribution to sepsis detection. These results agree with Kwon et al. (10) and Lui et al. (12), who also showed the suitability of XAI approaches to prioritize those SNPs that have a higher influence for phenotype prediction.

## *In silico* functional, clinical, and biological interpretation

### Functional analysis

Among the 20 SNPs with the highest SHAP contribution values, we found an intronic variant within the *PRIM2* gene (0.054), a missense variant (0.050) located in the *RBSN* gene, and another intronic variant (0.049) located in the *SYNPR* gene. In particular, based on the GTEx database, *PRIM2* is mainly expressed in fibroblasts and prostate, *SYNPR* in brain tissue, and *RBSN* in brain and artery. On the other hand, based on PhenoScanner, some SNPs were associated with other phenotypes (**Table S5 extended,** included as a supplemental excel file), but among the most relevant traits we observed white blood cell count (p=5.98x10^-3^, rs201088712), sepsis (p=1.38x10^-3^, rs3015358), inflammatory disorders of male genital organs (p=2.08x10^-4^, rs75858984) and sepsis-associated death (p=4.58x10^-6^, rs114065456). Likewise, querying HaploReg v4.2 we found evidence of multiple elements related to chromatin status (enhancer histone marks: H3K4me1, H3K27ac; and promoter histone marks: H3K4me3, H3K9ac), conservation, and alterations of regulatory motifs, as well as DNase I sensitivity quantitative trait loci in different cell lines of multiple variants of the top 20 SNPs. Among the specific cell lines, we observed cells related to liver or lung, as well as blood, fibroblast and immune response (**Table S5**). One variant from the top 20 SNPs has high (>15) Combined Annotation Dependent Depletion (CADD) score, suggesting a deleterious role of this potentially pathogenic variant (**Table S5**). Four SNPs (rs11205009, rs111474353, rs7393, and rs12811317) also showed significant quantitative trait loci (eQTLs) in artery tibial, cells cultured fibroblasts, lung, artery aorta, whole blood, among others (**Table S5**, **Table S5 extended**).

Of note, the SNP with the highest SHAP contribution score is located in the *PRIM2* gene, which encodes the large subunit of DNA primase, a key enzymatic component of DNA replication in eukaryotic cells (24). Since sepsis is characterized by multi-organ dysfunction resulting from processes such as oxidative stress, apoptosis and metabolic alterations, among others, cell proliferation and DNA replication are highly regulated processes (25). In fact, in a gene co-expression network analysis identified genes (MICU2, CCAR1, and FNTA) related to apoptosis within a gene module for septic shock patients (26). *PRIM2* has been related to consecutive trauma-induced sepsis based on an expression profiling analysis (27). Querying Gene2drug database (<https://gene2drug.tigem.it/>), 1261 significant drugs for the *PRIM2* gene were found, being the most significant drug an antifungal, miconazole (p=5.12x10^-15^). The second SNP with the highest SHAP contribution score was located in the *RBSN* gene, which encodes a protein belonging to the FYVE zinc finger family and has been linked to the MyD88-dependent cascade initiated by endosomal and innate immune pathways based on PathCards (<https://pathcards.genecards.org/>). These two pathways are essential during infection and sepsis. In particular, the zinc finger protein family has multiple functions, including transcriptional regulation, which influences in the gene expression, DNA repair, and cell differentiation (28–31). Zinc finger proteins may contribute to inflammation, immune cell function and tissue repair by modulating gene expression and may be involved in the regulation of pro-inflammatory cytokines, anti-inflammatory factors, and other immune-related genes critical for a balanced immune response during infection (30). Furthermore, since cellular dysfunction and apoptosis occur during sepsis, these proteins may be involved in the regulation of these processes to influence cell survival and tissue integrity, as well as in DNA repair mechanisms to mitigate the effects of oxidative stress (28). On the other hand, *SYNPR* encodes the protein synaptoporin, which is found in the central nervous system, particularly at synapses, and is involved in synaptic vesicle trafficking and neurotransmitter release (32).

### Clinical implications

**Table S6** summarizes the main implications of the top20 SNPs, while the excel file “Table S6 extended” shows all the results from association analysis. Among the top 20 SNPs, we found associations with comorbidities, measurements at diagnosis, sources of infection, and time course and hospital outcomes (*p*-value <0.05). Nonetheless, only rs79219127, intronic to the *FAM155A* gene, and rs79275514, intronic to the gene encoding the Parkin protein (*PARK2*), showed statistically significant associations after Bonferroni correction for multiple comparisons (*p*-value <0.0025). In detail, rs79219127 was significantly associated with the length of hospital (*p*-value=2.6x10^-8^) and ICU stay (*p*-value =7.7x10^-4^), while rs79275514 was significantly related to high blood pressure (*p*-value=2.1x10^-4^) and chronic liver failure (*p*-value=3.2x10^-5^). This is consistent with the fact that the top 20 SNPs were defined as those with the highest contribution towards sepsis prediction rather than towards clinical prognosis.

### Enrichment analysis

In terms of the most relevant enriched biological process, we identified the negative regulation of heart contraction (GO:0045822, p=6.31x10^-5^; adjusted p=0.02) (**Figure S7**). This process involves two genes, renalase, FAD dependent amine oxidase (*RNLS*), and phosphodiesterase 4D (*PDE4D*). This biological process is very interesting because cardiac dysfunction is an important consequence of sepsis that affects mortality, and this dysfunction is often attributed to factors such as increased inflammation or suppression of fatty acid and glucose oxidation, leading to eventual adenosine triphosphate (ATP) depletion (33).

Additionally, we observed that cyclic-nucleotide phosphodiesterase activity (GO:0004112, p=3.22x10^-5^; adjusted p=1.55x10^-3^), cyclic adenosine monophosphate (cAMP) binding (GO:0030552, p=6.31x10^-5^; adjusted p=1.55x10^-3^), and cyclic nucleotide binding (GO:0030551, p=2.40x10^-4^; adjusted p=3.91x10^-3^) were the most significantly enriched molecular processes (**Figure S8**). These processes involve two phosphodiesterase genes (*PDE10A* and *PDE4D*). Phosphodiesterases play an essential role in modulating of cyclic nucleotide signaling (cAMP and cyclic guanosine monophosphate (cGMP)) and have a wide range of physiological functions (34,35). In particular, cAMP and cGMP are involved in several cellular processes, including immune response and inflammation (36,37). Moreover, phosphodiesterase inhibitors have shown potential therapeutic effects in experimental models of sepsis and lung inflammation (38–41). By inhibiting phosphodiesterase enzymes, cAMP and cGMP levels increased, which modulate immune cell function and leading to immune modulation and anti-inflammatory effects (38,39). A recent GWAS of sepsis risk identified variants located in genes of the same family (*PDE4B*, *PDE4A,* and *PDE7A*) that were also associated with mortality (42).

Concerning enrichment in the Jessen disease database, acrodysostosis (p=5.49x10^-3^; adjusted p=0.068) and dementia (p=7.50x10^-3^; adjusted p=0.068) emerged as the most relevant diseases. These conditions involve the genes *PDE4D*, Sortilin Related Receptor 1 (*SORL1*), and Parkin RBR E3 Ubiquitin Protein Ligase (*PARK2*) (**Figure S9**). In this case, *PARK2* has been described as an E3 ligase involved in the regulation of cardiac mitophagy, which is important for the maintenance of normal cardiac mitochondrial function (43–45). Since mitochondrial dysfunction and autophagy, as well as cardiac dysfunction, are activated during sepsis, a study in mice suggested a possible additional protective role of *PARK2* in mitochondria, as autophagic clearance of damaged mitochondria still occurred, suggesting compensatory mechanisms involving *PARK2*-independent mitophagy and upregulation of macroautophagy (46,47). On the other hand, *SORL1* is involved in the trafficking and processing of the amyloid precursor protein (APP), and its dysfunction has been implicated in the pathogenesis of Alzheimer's disease and neurodegenerative disorders (48,49).

In the expression heatmap across different tissues, we observed that *RBSN,* as well as, Lysophospholipase Like 1 (*LYPLAL1*), and Zinc Finger Protein 775 (*ZNF775*) genes were highly expressed in almost all the different tissues (**Figure S10**). Likewise, *SORL1, SYNPR,* and ST8 Alpha-N-Acetyl-Neuraminide Alpha-2,8-Sialyltransferase 3 *(ST8SIA3*) genes are highly expressed especially in brain tissues (**Figure S10**).

# Supplemental Tables

**Table S1**. **Sepsis prediction performance in the training set with the CNN models obtained with each input SNP subset.**

| ***p*-value threshold** | **Number of SNPs selected** | **Performance metrics** | | | | |
| --- | --- | --- | --- | --- | --- | --- |
|  |  | **Se (%)** | **Sp (%)** | **Acc (%)** | **AUC** | **OR** |
| 5*10^-2^ | 33596 | 100 | 100 | 100 | 1 | N.D |
| **5*10^-3^** | **3761** | **100** | **100** | **100** | **1** | **N.D** |
| 5*10^-4^ | 495 | 99.7 | 99.9 | 99.9 | 0.999 | 6.6e+5 |
| 5*10^-5^ | 100 | 88.0 | 99.5 | 97.5 | 0.998 | 1.4e+3 |
| 5*10^-6^ | 37 | 76.6 | 99.7 | 95.6 | 0.986 | 950.73 |
| 5*10^-7^ | 30 | 69.0 | 99.8 | 94.5 | 0.963 | 1.0e+3 |
| 5*10^-8^ | 28 | 69.1 | 99.7 | 94.3 | 0.954 | 781.79 |

*Abbreviations*: Acc: accuracy; AUC: area under the receiver operating characteristic (ROC) curve; N.D: not defined; OR: odds ratio; Se: sensitivity, Sp: specificity; SNP: single-nucleotide polymorphism

**Table S2**. **Sepsis prediction performance in the validation set with the CNN models obtained with each input SNP subset.**

| ***p*-value threshold** | **Number of SNPs selected** | **Performance metrics** | | | | |
| --- | --- | --- | --- | --- | --- | --- |
|  |  | **Se (%)** | **Sp (%)** | **Acc (%)** | **AUC** | **OR** |
| 5*10^-2^ | 33596 | 70.6 | 99.3 | 94.3 | 0.977 | 347.60 |
| **5*10^-3^** | **3761** | **79.7** | **98.1** | **94.8** | **0.972** | **197.90** |
| 5*10^-4^ | 495 | 80.2 | 97.4 | 94.4 | 0.962 | 150.18 |
| 5*10^-5^ | 100 | 71.7 | 98.4 | 93.7 | 0.952 | 155.49 |
| 5*10^-6^ | 37 | 68.5 | 98.4 | 93.1 | 0.919 | 133.42 |
| 5*10^-7^ | 30 | 66.3 | 99.0 | 93.2 | 0.914 | 189.39 |
| 5*10^-8^ | 28 | 63.6 | 99.0 | 92.8 | 0.904 | 168.39 |

*Abbreviations*: Acc: accuracy; AUC: area under the receiver operating characteristic (ROC) curve; N.D: not defined; OR: odds ratio; Se: sensitivity, Sp: specificity; SNP: single-nucleotide polymorphism

**Table S3**. **Sepsis prediction performance in the test set with the CNN models obtained with each input SNP subset.**

| ***p*-value threshold** | **Number of SNPs selected** | **Performance metrics** | | | | |
| --- | --- | --- | --- | --- | --- | --- |
|  |  | **Se (%)** | **Sp (%)** | **Acc (%)** | **AUC** | **OR** |
| 5*10^-2^ | 33596 | 65.8 | 99.3 | 93.4 | 0.973 | 278.67 |
| **5*10^-3^** | **3761** | **85.6** | **98.7** | **96.4** | **0.985** | **465.99** |
| 5*10^-4^ | 495 | 80.8 | 96.8 | 94.0 | 0.962 | 127.03 |
| 5*10^-5^ | 100 | 76.5 | 97.8 | 94.1 | 0.955 | 146.59 |
| 5*10^-6^ | 37 | 69.0 | 98.6 | 93.4 | 0.937 | 160.14 |
| 5*10^-7^ | 30 | 66.8 | 99.1 | 93.4 | 0.928 | 218.75 |
| 5*10^-8^ | 28 | 63.6 | 99.0 | 92.8 | 0.913 | 168.58 |

*Abbreviations*: Acc: accuracy; AUC: area under the receiver operating characteristic (ROC) curve; N.D: not defined; OR: odds ratio; Se: sensitivity, Sp: specificity; SNP: single-nucleotide polymorphism

**Table S4**. **Sepsis prediction performance in the test set with the CNN models obtained with each number of top SNP selected.**

| Number of Top SNPs selected | Performance metrics | | | | |
| --- | --- | --- | --- | --- | --- |
|  | Se (%) | Sp (%) | Acc (%) | AUC | OR |
| **3761** | **85.6** | **98.7** | **96.4** | **0.985** | **465.99** |
| 1,000 | 82.9 | 98.5 | 95.8 | 0.981 | 321.55 |
| 500 | 80.2 | 98.2 | 95.0 | 0.972 | 217.91 |
| 100 | 79.1 | 97.2 | 94.0 | 0.958 | 129.18 |
| 50 | 72.7 | 98.3 | 93.8 | 0.952 | 108.21 |
| **20** | **75.9** | **98.5** | **94.5** | **0.951** | **209.48** |
| 10 | 74.9 | 95.9 | 92.2 | 0.939 | 69.50 |
| 5 | 71.7 | 95.4 | 91.3 | 0.926 | 52.84 |
| 4 | 71.1 | 95.6 | 91.4 | 0.921 | 55.85 |
| **3** | **70.1** | **94.5** | **90.2** | **0.886** | **40.35** |
| 2 | 46.0 | 98.5 | 89.3 | 0.771 | 56.53 |

*Abbreviations*: Acc: accuracy; AUC: area under the receiver operating characteristic (ROC) curve; OR: odds ratio; Se: sensitivity, Sp: specificity; SNP: single-nucleotide polymorphism

| **Table S5**. **Functional assessment of Top 20 variants with the highest SHAP contribution values.** | | | | | | | |
| --- | --- | --- | --- | --- | --- | --- | --- |
| **SNPs**  **(Nearest genes)** | **RegulomedB rank (score)** | **eQTLs** [GTEx]  **Tissue-specific p ≤ 0.05** | **V2G** | **CADD**  **(>15 in bold)** | **Enhancer histone marks**  **(H3K4me1, H3K27ac)**  [HaploReg] | **Promoter histone marks**  **(H3K4me3, H3K9ac)** [HaploReg] | **DNAse**  [HaploReg] |
| rs1018040  (*TGFB2/LYPLAL1*) | TF binding or DNase peak (5, 0.0) | None | 0.060 (*RRP15*) | 6.58 | See Table S5 extended | See Table S5 extended | None |
| rs11205009  (*LCE5A*) | eQTL + TF binding/Dnase peak (1f, 0.55436) | 32 tissues (Artery Tibial (p=1.10E-06); Lung (p=2.70E-07); Cells - Cultured fibroblasts (p=5.30E-07); Heart Left Ventricle (p=1.50E-05); Heart Atrial Appendage (p=4.10E-05); See Table S5 extended) | 0.126 (*FLG*) | 5.91 | Primary T cells from peripheral blood; Primary T cells effector/memory enriched from peripheral blood; Primary T helper cells from peripheral blood; Primary T helper cells PMA-I stimulated; Primary T helper 17 cells PMA-I stimulated; Primary T helper memory cells from peripheral blood 2; Primary T helper naive cells from peripheral blood; Primary T CD8+ naive cells from peripheral blood; Primary Natural Killer cells from peripheral blood; Esophagus; Liver; See Table S5 extended | Primary T helper naive cells from peripheral blood; Liver; See Table S5 extended | See Table S5 extended |
| **rs1575081785 (*RBSN*)** | **NA** | **NA** | **NA** | **NA** | **None** | **None** | **None** |
| **rs74707084**  **(*SYNPR*)** | **TF binding + DNase peak (4, 0.60906)** | **None** | **0.060 (*SYNPR*)** | **19.2** | **See Table S5 extended** | **None** | **See Table S5 extended** |
| rs1979271  (*RBMS3*) | Other  (7, 0.18412) | None | 0.020 (*RBMS3*) | 1.5 | Primary T helper naive cells from peripheral blood; See Table S5 extended | See Table S5 extended | None |
| rs62372011  (*PDE4D*) | TF binding or DNase peak (5, 0.13454) | None | 0.080 (*PDE4D*) | 6.24 | Aorta | See Table S5 extended | None |
| **rs17653532**  **(*PRIM2*)** | **TF binding + any motif + DNase peak (3a, 0.29787)** | **None** | **0.093 (*BAG2*)** | **14.3** | **Primary T helper cells PMA-I stimulated; Primary T CD8+ naive cells from peripheral blood; NHLF Lung Fibroblast Primary Cells; Primary T helper cells PMA-I stimulated; See Table S5 extended** | **Primary T CD8+ naive cells from peripheral blood; See Table S5 extended** | **None** |
| rs79275514  (*PARK2*) | Motif hit  (6, 0.49716) | None | 0.020 (*PRKN*) | 2.5 | See Table S5 extended | Monocytes-CD14+ RO01746 Primary Cells; See Table S5 extended | None |
| rs111474353 (*DPCR1*) | TF binding or DNase peak (5, 0.13454) | 8 tissues (Artery Tibial (p=1.00E-05); See Table S5 extended) | 0.127 (*HLA-C*) | 1.42 | Lung; See Table S5 extended | See Table S5 extended | None |
| rs903439  (*PDE10A*) | Other  (7, 0.18412) | None | 0.053 (*PDE10A*) | 3.71 | See Table S5 extended | See Table S5 extended | None |
| rs7393  (*ZNF775*) | eQTL + TF binding/Dnase peak (1f, 0.55436) | 28 tissues (Whole Blood (p=5.10E-05); Cells Cultured fibroblasts (p=8.40E-06); Lung (p=1.80E-07); Heart Left Ventricle (p=4.00E-07); Heart Atrial Appendage (p=6.10E-05); Artery Tibial (p=6.20E-05); See Table S5 extended) | 0.287 (*ZNF775*) | 5.71 | Primary T cells from peripheral blood; Foreskin Fibroblast Primary Cells skin01; Foreskin Fibroblast Primary Cells skin02; Right Ventricle; See Table S5 extended | See Table S5 extended | None |
| rs10887816  (*RNLS*) | TF binding or DNase peak (5, 0.00454) | None | 0.067 (*RNLS*) | 3.8 | See Table S5 extended | See Table S5 extended | See Table S5 extended |
| rs77119441 (*SORL1*) | Other  (7, 0.18412) | None | 0.040 (*BLID*) | 2.9 | See Table S5 extended | None | None |
| rs12811317 (*SYT10*) | eQTL + TF binding/Dnase peak (1f, 0.83785) | Cells - Cultured fibroblasts (p=3.90E-04) | 0.066 (*SYT10*) | 0.648 | See Table S5 extended | None | None |
| rs79219127 (*FAM155A*) | Motif hit  (6, 0.5804) | None | 0.027 (*NALF1*) | 6.00 | None | None | None |
| rs201088712 (*CENPJ*) | TF binding + DNase peak (4, 0.60906) | NA | NA | NA | Aorta; Foreskin Fibroblast Primary Cells skin01; Monocytes-CD14+ RO01746 Primary Cells; See Table S5 extended | None | None |
| rs3015358 (*DAOA-AS1*) | Other  (7, 0.18412) | None | 0.033 (*DAOA*) | 3.81 | None | See Table S5 extended | None |
| rs114065456 (*FLRT2/LINC01148)* | Other  (7, 0.18412) | None | NA | 1.67 | None | See Table S5 extended | None |
| rs4028683 (*NPAP1*) | Other  (7, 0.18412) | None | 0.060 (*SNRPN*) | 0.132 | None | None | None |
| rs75858984 (*ST8SIA3*) | Other  (7, 0.18412) | None | 0.066 (*ST8SIA3*) | 1.23 | None | See Table S5 extended | None |
| CADD, Combined annotation dependent depletion; DNase I sensitivity quantitative trait loci; eQTL, expression quantitative trait loci; Open Targets Genetics (Top ranked genes based on the overall V2G score: assigning variants to genes). Only interesting cells (immunity, blood, heart, liver, lung and kidney) are shown. See more complete information in the Table S5 extended (included as a supplementary excel file). | | | | | | | |

**Table S6**. **Main clinical implications of the top 20 SNPs for sepsis prediction.**

|  | N^a^ | Associated SNPs^b^ |
| --- | --- | --- |
| Comorbidities |  |  |
| Chronic cardiovascular disease | 730 | rs1575081785, rs114065456 |
| Chronic respiratory disease | 737 | rs114065456 |
| High blood pressure | 737 | **rs79275514^c^** |
| Chronic renal failure | 737 | rs114065456 |
| Chronic liver failure | 730 | **rs79275514^c^**, rs12811317 |
| Diabetes mellitus | 737 | - |
| Obesity | 730 | rs114065456 |
| Immunosuppression | 737 | rs79275514 |
| Measurements at diagnosis |  |  |
| Creatinine | 750 | rs903439 |
| White Blood cells | 750 | rs1018040, rs903439, rs12811317 |
| Lymphocytes | 750 | rs1979271 |
| Neutrophils | 750 | rs7393, rs114065456 |
| SOFA score | 750 | - |
| APACHE II score | 750 | rs62372011 |
| Source of infection |  |  |
| Pneumonia | 740 | - |
| Peritonitis | 740 | - |
| Catheter | 740 | rs1575081785, rs77119441 |
| Surgical site | 740 | rs1575081785, rs77119441 |
| Time course and hospital outcomes |  |  |
| Length of hospital stay | 748 | rs1575081785, rs111474353, rs77119441, rs12811317, **rs79219127^c^** |
| Length of ICU stay | 747 | rs1979271, rs74707084, rs111474353, **rs79219127^c^** |
| Length of mechanical ventilation | 744 | rs79275514, rs79219127, rs114065456 |
| Septic shock | 750 | rs79219127, rs4028683 |
| Mortality at 90 days | 750 | rs62372011 |

*Abbreviations*: SOFA: Sequential Organ Failure Assessment; APACHE II: Acute Physiology And Chronic Health Evaluation II; SNP: single nucleotide polymorphism; ICU: intensive care unit.

^a^ N: number of subjects with information of the clinical variable (i.e., not missing value)

^b^ *p*-value <0.05.

^c^ *p*-value < 0.0025 (0.05/20, Bonferroni correction).

See complete association results in the Table S6 extended, included as a supplementary excel file.

# Supplemental Figures

**
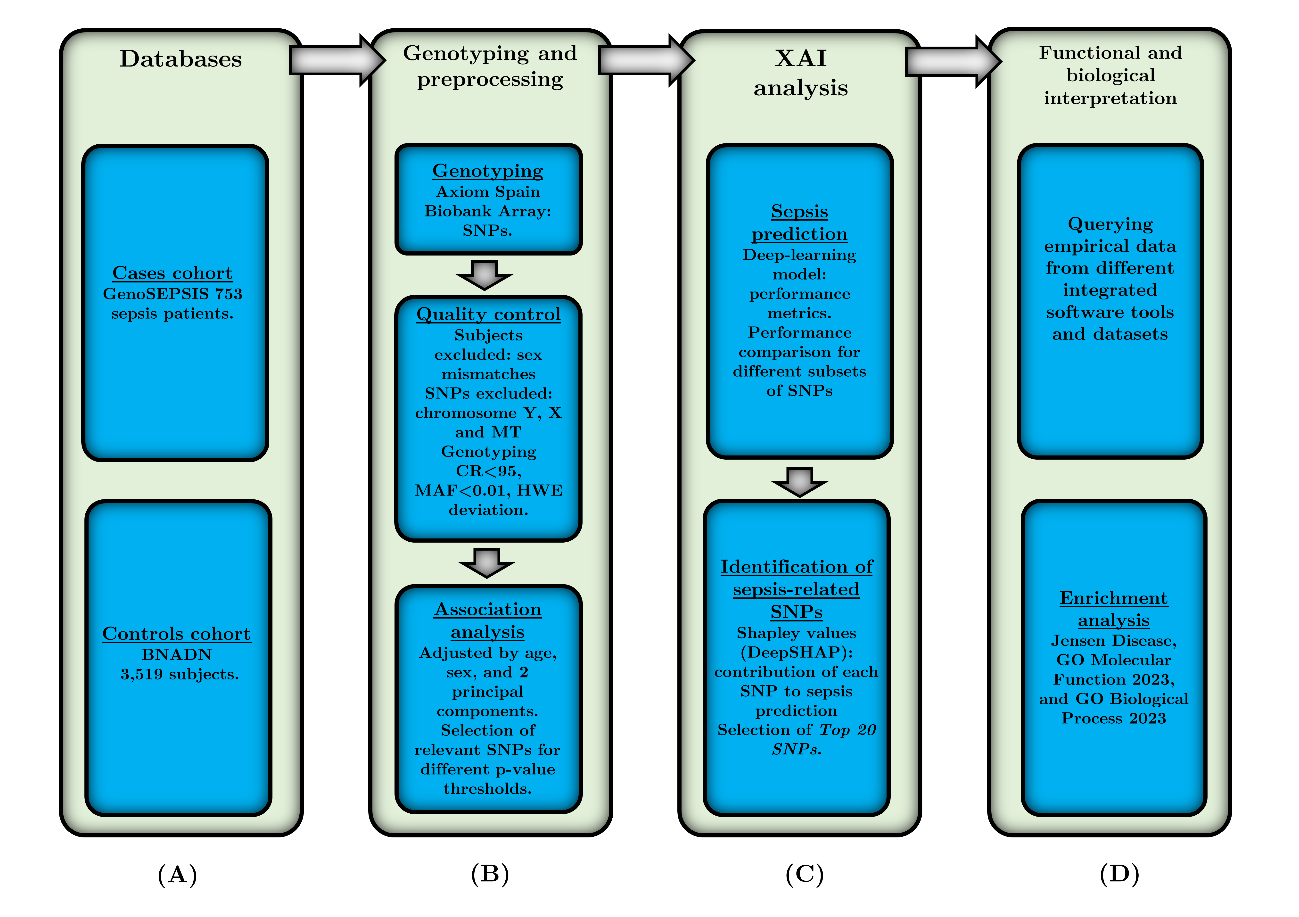
**

**Figure S1.** **Flowchart of the proposed methodology**. (**A**) Databases. (**B**) Genotyping and preprocessing. (**C**) XAI analysis. (**D**) Functional and biological interpretation.

**Figure S2.** **Principal component analysis**. Plot of the first two principal components (PCs) of genetic variation of cases and controls individuals analyzed, projected on the HapMap3 reference dataset.


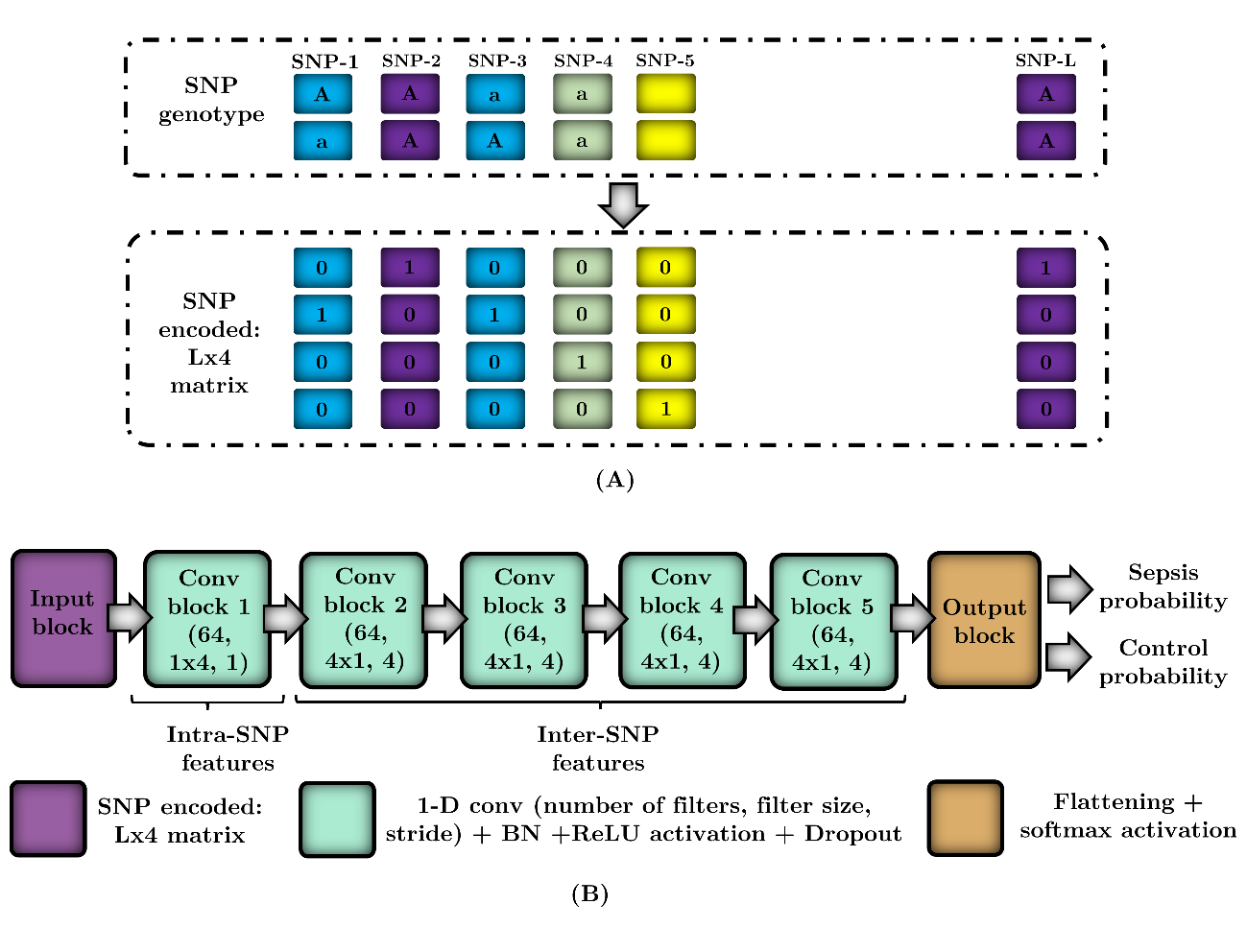


**Figure S3.** **Overview of the proposed CNN-based architecture for sepsis prediction.** (**A**) Encoding of the input SNP data into a one-hot representation. (**B**) Processing of the SNP encoded through a CNN architecture.


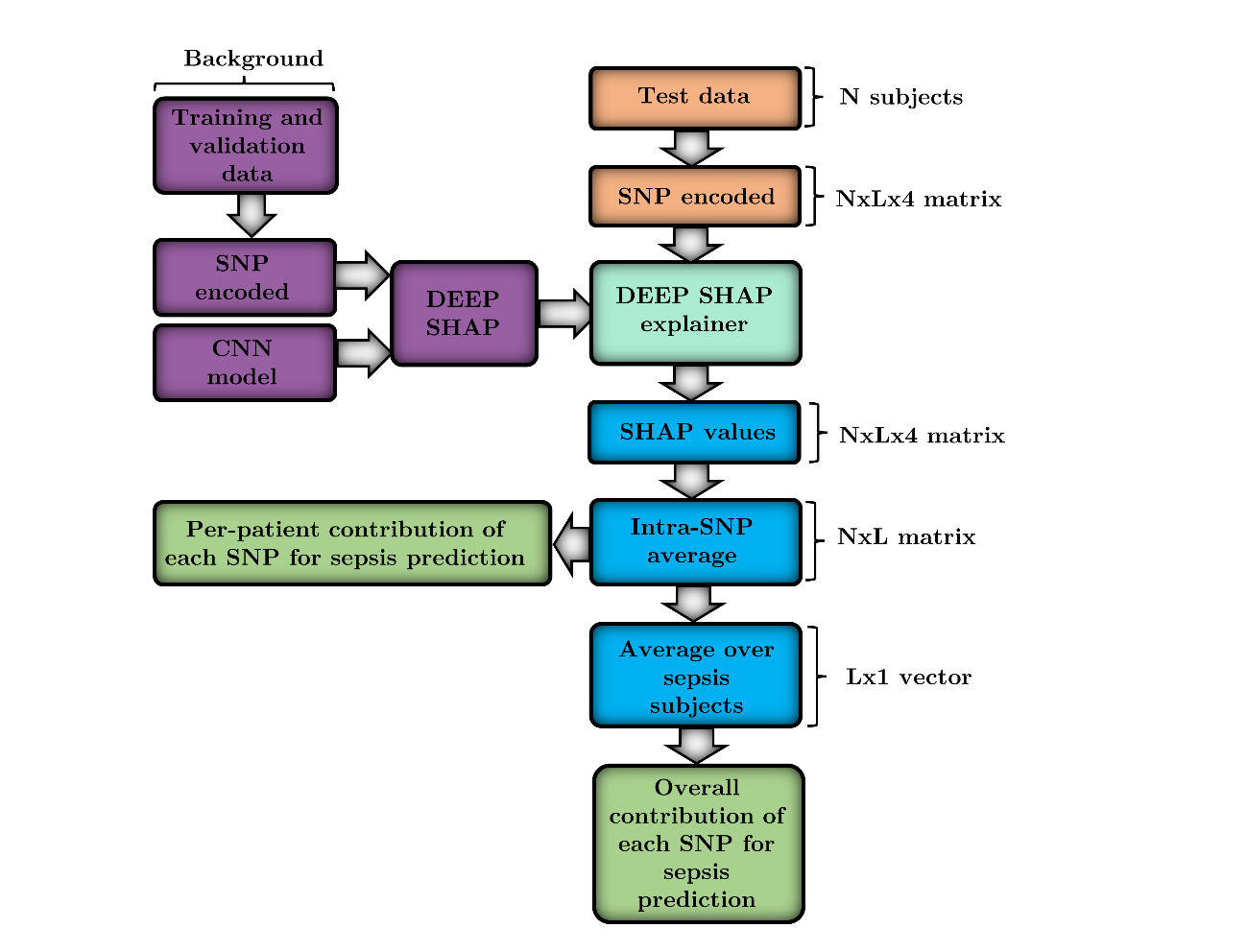


**Figure S4.** **Overview of the proposed Deep SHAP approach for identifying sepsis-related SNPs**.


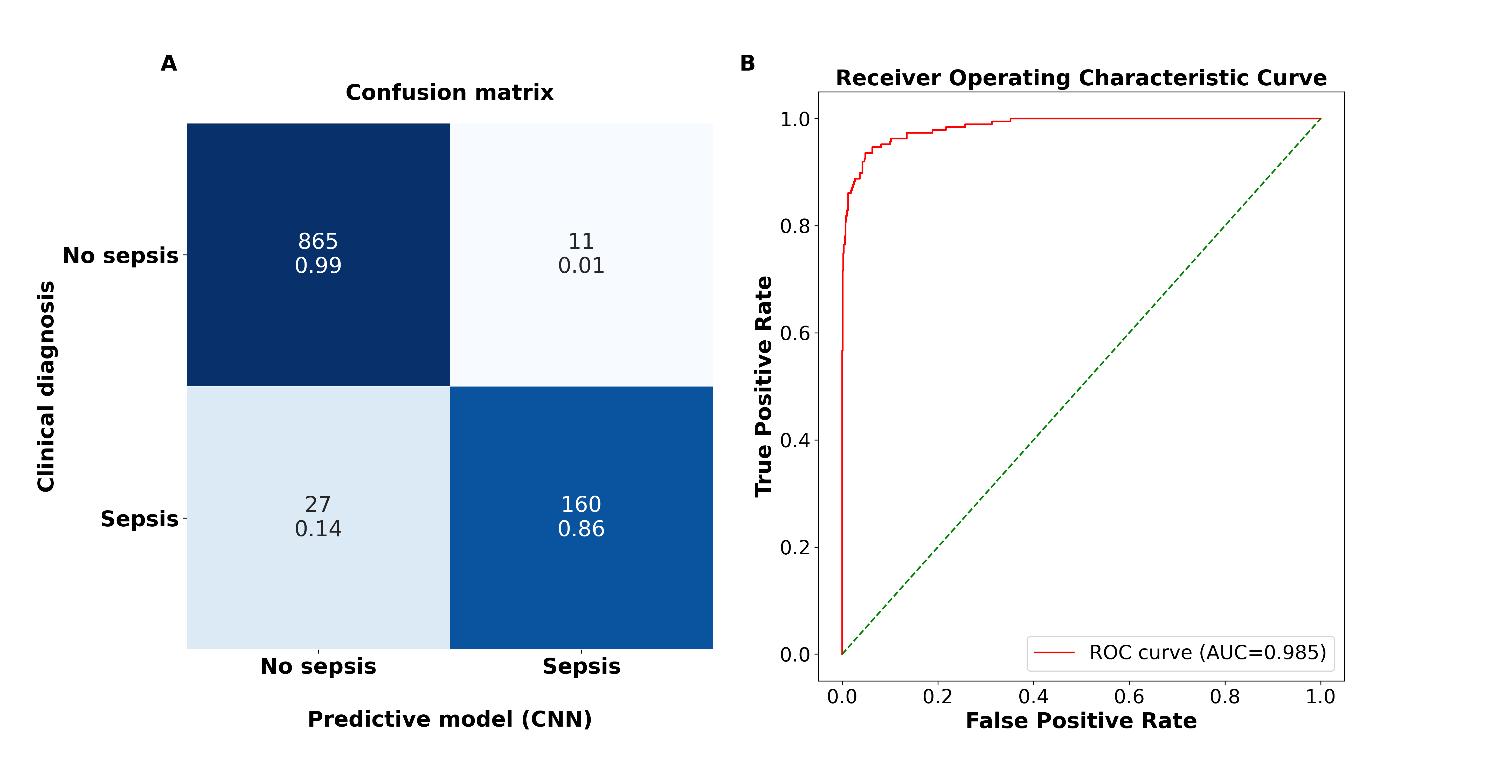


**Figure S5.** **Sepsis prediction performance of the best CNN model in the test set.** (**A**) Confusion matrix of CNN-based deep-learning model for binary sepsis classification. (**B**) Receiver operating characteristics (ROC) curve of the CNN model.


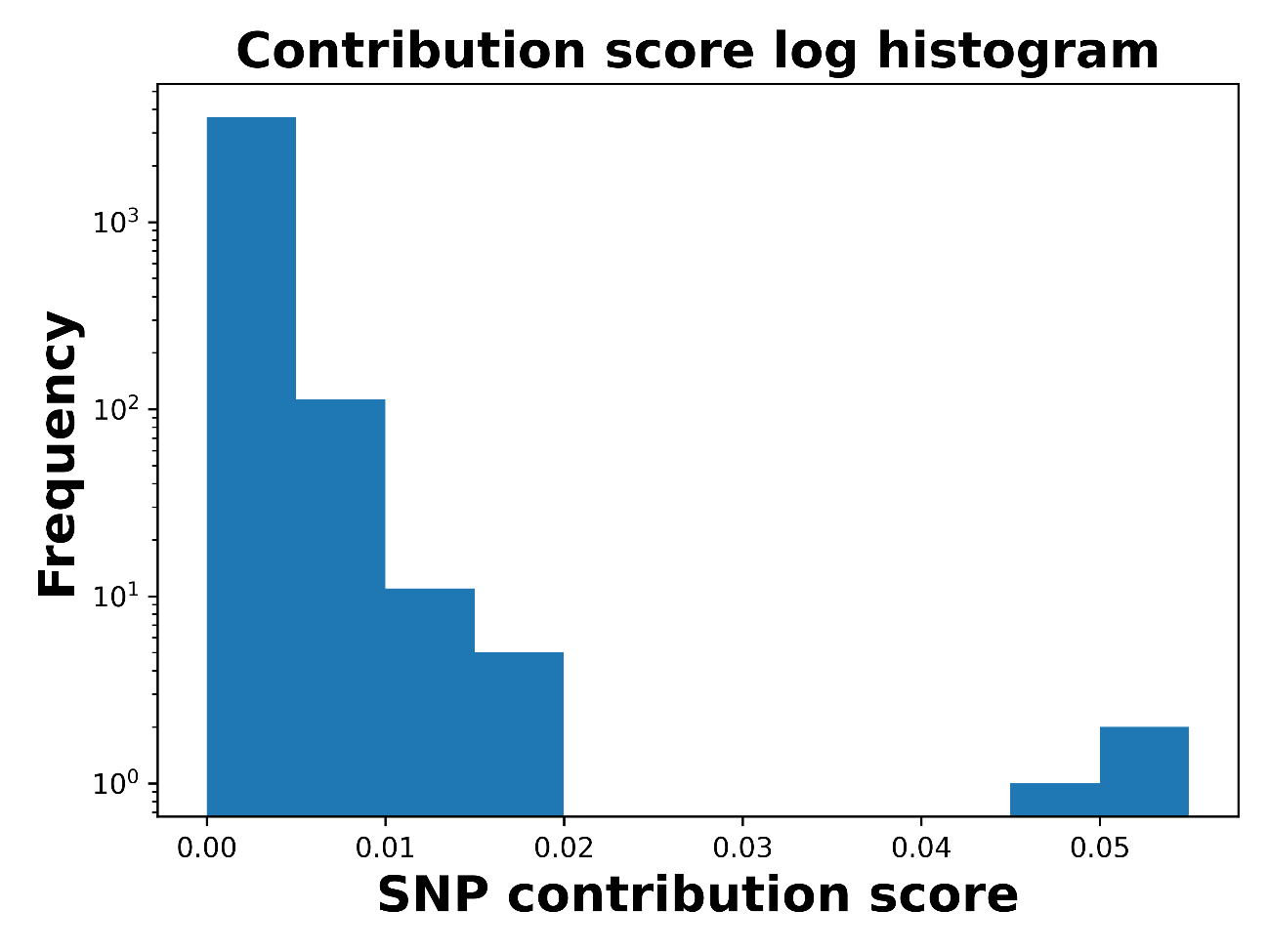


**Figure S6.** **Log histogram of the SNP contribution scores for sepsis prediction**.


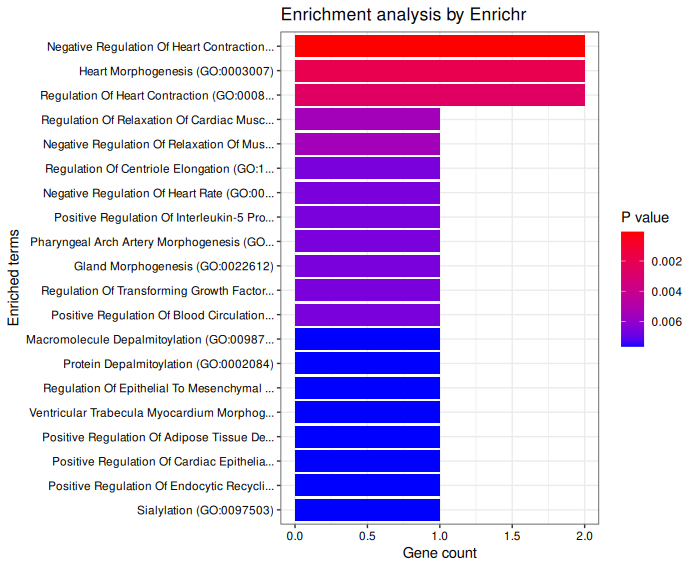


**Figure S7.** **Gene Ontology (GO) enrichment analysis for related genes from the top 20 SNPs querying GO Biological Process 2023.**


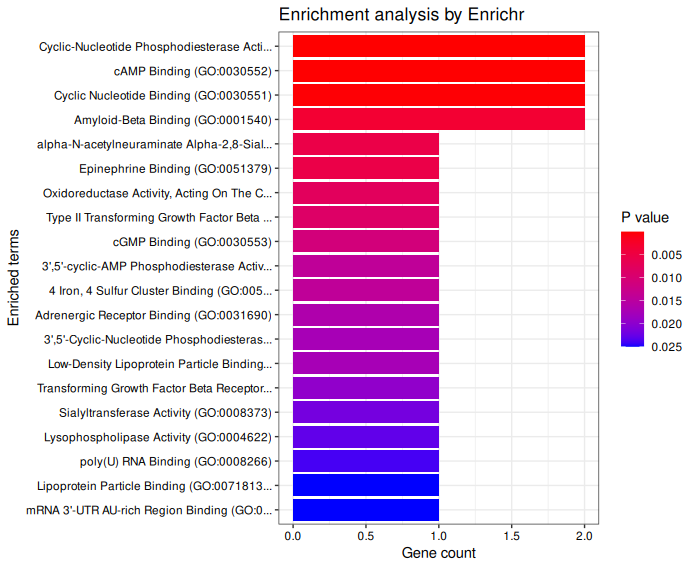


**Figure S8.** **Gene Ontology (GO) enrichment analysis for related genes from the top 20 SNPs querying GO Molecular Function 2023.**


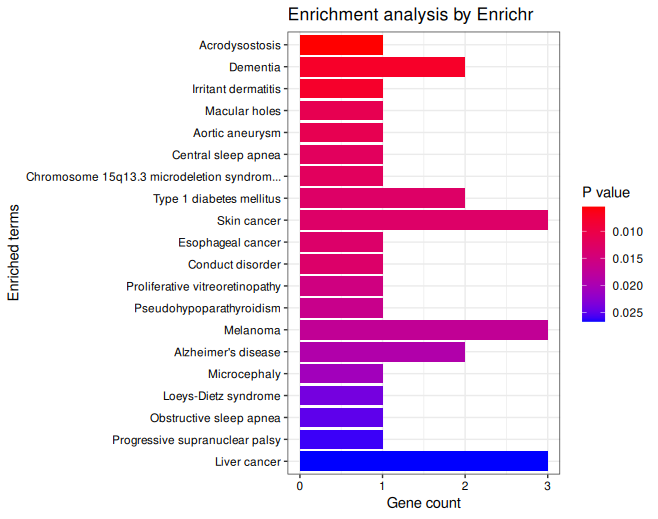


**Figure S9.** **Gene Ontology (GO) enrichment analysis for related genes from the top 20 SNPs querying Jensen Disease database.**

**
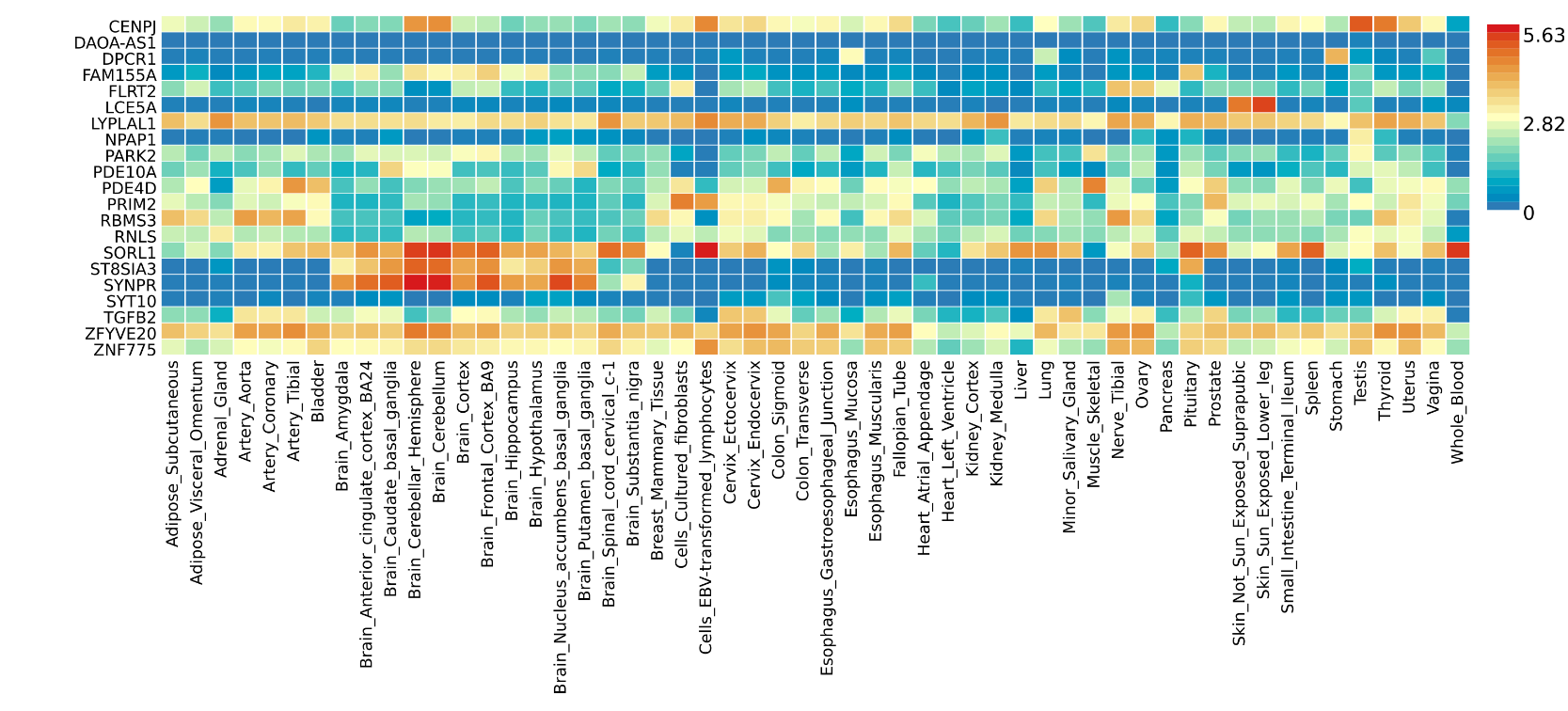
**

**Figure S10.** **Expression heatmap of the related genes across different tissues.**

# Supplemental References

1. Singer M, Deutschman CS, Seymour C, Shankar-Hari M, Annane D, Bauer M, Bellomo R, Bernard GR, Chiche JD, Coopersmith CM, et al. The third international consensus definitions for sepsis and septic shock (sepsis-3). *JAMA - J Am Med Assoc* (2016) 315:801–810. doi: 10.1001/jama.2016.0287

2. Levy MM, Evans LE, Rhodes A. The Surviving Sepsis Campaign Bundle: 2018 update. *Intensive Care Med* (2018) 44:925–928. doi: 10.1007/s00134-018-5085-0

3. Martín-Fernández M, Heredia-Rodríguez M, González-Jiménez I, Lorenzo-López M, Gómez-Pesquera E, Poves-Álvarez R, Álvarez FJ, Jorge-Monjas P, Beltrán-DeHeredia J, Gutiérrez-Abejón E, et al. Hyperoxemia in postsurgical sepsis/septic shock patients is associated with reduced mortality. *Crit Care* (2022) 26:1–9. doi: 10.1186/s13054-021-03875-0

4. Vincent J-L, Moreno R, Takala J, Willatts S, De Mendonça A, Bruining H, Reinhart CK, Suter P, Thijs LG. The SOFA (Sepsis-related Organ Failure Assessment) score to describe organ dysfunction/failure: On behalf of the Working Group on Sepsis-Related Problems of the European Society of Intensive Care Medicine (see contributors to the project in the appendix). *Intensive Care Med* (1996) 22:707–710.

5. Knaus WA, Draper EA, Wagner DP, Zimmerman JE. APACHE II: a severity of disease classification system. *Crit Care Med* (1985) 13:818–829.

6. Cruz R, Diz-de Almeida S, López de Heredia M, Quintela I, Ceballos FC, Pita G, Lorenzo-Salazar JM, González-Montelongo R, Gago-Domínguez M, Sevilla Porras M, et al. Novel genes and sex differences in COVID-19 severity. *Hum Mol Genet* (2022) 31:3789–3806. doi: 10.1093/hmg/ddac132

7. Suarez-Pajes E, Marcelino-Rodriguez I, Hernández Brito E, Gonzalez-Barbuzano S, Ramirez-Falcon M, Tosco-Herrera E, Rubio-Rodríguez LA, Briones ML, Rajas O, Borderías L, et al. A genome-wide association study of adults with community-acquired pneumonia. *Respir Res* (2024) 25:374. doi: 10.1186/s12931-024-03009-4

8. Chang CC, Chow CC, Tellier LCAM, Vattikuti S, Purcell SM, Lee JJ. Second-generation PLINK: rising to the challenge of larger and richer datasets. *Gigascience* (2015) 4:s13742--015.

9. Lecun Y, Bengio Y, Hinton G. Deep learning. *Nature* (2015) 521:436–444. doi: 10.1038/nature14539

10. Kwon OS, Hong M, Kim TH, Hwang I, Shim J, Choi EK, Lim HE, Yu HT, Uhm JS, Joung B, et al. Genome-wide association study-based prediction of atrial fibrillation using artificial intelligence. *Open Hear* (2022) 9:1–10. doi: 10.1136/openhrt-2021-001898

11. Mieth B, Rozier A, Rodriguez JA, Höhne MMC, Görnitz N, Müller KR. DeepCOMBI: Explainable artificial intelligence for the analysis and discovery in genome-wide association studies. *NAR Genomics Bioinforma* (2021) 3:1–21. doi: 10.1093/nargab/lqab065

12. Liu L, Feng X, Li H, Cheng Li S, Qian Q, Wang Y. Deep learning model reveals potential risk genes for ADHD, especially Ephrin receptor gene EPHA5. *Brief Bioinform* (2021) 22:1–11. doi: 10.1093/bib/bbab207

13. Goodfellow I, Bengio Y, Courville A. *Deep Learning*. MIT Press. (2016).

14. Yang G, Ye Q, Xia J. Unbox the black-box for the medical explainable AI via multi-modal and multi-centre data fusion: A mini-review, two showcases and beyond. *Inf Fusion* (2022) 77:29–52. doi: 10.1016/j.inffus.2021.07.016

15. Lundberg SM, Lee S-I. A Unified Approach to Interpreting Model Predictions. *31st Conference on Neural Information Processing Systems (NIPS 2017),*. (2017). p. 1208–1217 https://doi.org/10.1016/j.inffus.2019.12.012%0Ahttps://doi.org/10.1016/j.ophtha.2018.11.016

16. Withnell E, Zhang X, Sun K, Guo Y. XOmiVAE: an interpretable deep learning model for cancer classification using high-dimensional omics data. *Brief Bioinform* (2021) 22:1–11. doi: 10.1093/bib/bbab315

17. Ward LD, Kellis M. HaploReg: a resource for exploring chromatin states, conservation, and regulatory motif alterations within sets of genetically linked variants. *Nucleic Acids Res* (2012) 40:D930--D934.

18. Boyle AP, Hong EL, Hariharan M, Cheng Y, Schaub MA, Kasowski M, Karczewski KJ, Park J, Hitz BC, Weng S, et al. Annotation of functional variation in personal genomes using RegulomeDB. *Genome Res* (2012) 22:1790–1797.

19. Peat G, Jones W, Nuhn M, Marugán JC, Newell W, Dunham I, Zerbino D. The open targets post-GWAS analysis pipeline. *Bioinformatics* (2020) 36:2936–2937.

20. Lonsdale J, Thomas J, Salvatore M, Phillips R, Lo E, Shad S, Hasz R, Walters G, Garcia F, Young N, et al. The genotype-tissue expression (GTEx) project. *Nat Genet* (2013) 45:580–585.

21. Kuleshov M V, Jones MR, Rouillard AD, Fernandez NF, Duan Q, Wang Z, Koplev S, Jenkins SL, Jagodnik KM, Lachmann A, et al. Enrichr: a comprehensive gene set enrichment analysis web server 2016 update. *Nucleic Acids Res* (2016) 44:W90--W97.

22. Watanabe K, Taskesen E, Van Bochoven A, Posthuma D. Functional mapping and annotation of genetic associations with FUMA. *Nat Commun* (2017) 8:1826.

23. Watanabe K, Umićević Mirkov M, de Leeuw CA, van den Heuvel MP, Posthuma D. Genetic mapping of cell type specificity for complex traits. *Nat Commun* (2019) 10:3222.

24. Chung J, Tsai S, James AH, Thames BH, Shytle S, Piedrahita JA. Lack of genomic imprinting of DNA primase, polypeptide 2 (PRIM2) in human term placenta and white blood cells. *Epigenetics* (2012) 7:429–431.

25. Pober JS. Effects of tumour necrosis factor and related cytokines on vascular endothelial cells. *Ciba Foundation Symposium 131-Tumour Necrosis Factor and Related Cytotoxins: Tumour Necrosis Factor and Related Cytotoxins: Ciba Foundation Symposium 131*. (2007). p. 170–191

26. Martínez-Paz P, Gomez-Pilar J, Martín-Fernández M, Ceballos FC, Gómez-Sánchez E, Hornero R, Tamayo E. Gene Co-Expression Networks Offer New Perspectives on Sepsis Pathophysiology. *IEEE/ACM Trans Comput Biol Bioinforma* (2023) 20:3660–3668. doi: 10.1109/TCBB.2023.3309998

27. Dong L, Li H, Zhang S, Su L. Identification of genes related to consecutive trauma-induced sepsis via gene expression profiling analysis. *Medicine (Baltimore)* (2018) 97:

28. Maruyama K, Kidoya H, Takemura N, Sugisawa E, Takeuchi O, Kondo T, Eid MMA, Tanaka H, Martino MM, Takakura N, et al. Zinc finger protein St18 protects against septic death by inhibiting VEGF-A from macrophages. *Cell Rep* (2020) 32:

29. Cassandri M, Smirnov A, Novelli F, Pitolli C, Agostini M, Malewicz M, Melino G, Raschellà G. Zinc-finger proteins in health and disease. *Cell death Discov* (2017) 3:1–12.

30. Rakhra G, Rakhra G. Zinc finger proteins: insights into the transcriptional and post transcriptional regulation of immune response. *Mol Biol Rep* (2021) 48:5735–5743.

31. Liu X, Zhang P, Bao Y, Han Y, Wang Y, Zhang Q, Zhan Z, Meng J, Li Y, Li N, et al. Zinc finger protein ZBTB20 promotes Toll-like receptor-triggered innate immune responses by repressing I$κ$B$α$ gene transcription. *Proc Natl Acad Sci* (2013) 110:11097–11102.

32. Knaus P, Marquèze-Pouey B, Scherer H, Betzt H. Synaptoporin, a novel putative channel protein of synaptic vesicles. *Neuron* (1990) 5:453–462.

33. Zaky A, Deem S, Bendjelid K, Treggiari MM. Characterization of cardiac dysfunction in sepsis: an ongoing challenge. *Shock* (2014) 41:12–24.

34. Maurice DH, Ke H, Ahmad F, Wang Y, Chung J, Manganiello VC. Advances in targeting cyclic nucleotide phosphodiesterases. *Nat Rev Drug Discov* (2014) 13:290–314.

35. Bender AT, Beavo JA. Cyclic nucleotide phosphodiesterases: molecular regulation to clinical use. *Pharmacol Rev* (2006) 58:488–520.

36. Raker VK, Becker C, Steinbrink K. The cAMP pathway as therapeutic target in autoimmune and inflammatory diseases. *Front Immunol* (2016) 7:123.

37. Moore AR, Willoughby DA. The role of cAMP regulation in controlling inflammation. *Clin Exp Immunol* (1995) 101:387.

38. Hsu CG, Fazal F, Rahman A, Berk BC, Yan C. Phosphodiesterase 10A is a key mediator of lung inflammation. *J Immunol* (2021) 206:3010–3020.

39. Zhang Z, Liang M, Wan X. Roflumilast, a type of phosphodiesterase 4 inhibitor, can reduce intestinal injury caused by sepsis. *Exp Ther Med* (2021) 22:1–8.

40. Kazmi I, Al-Abbasi FA, Afzal M, Nadeem MS, Altayb HN, Gupta G. Phosphodiesterase-4 Inhibitor Roflumilast-Mediated Protective Effect in Sepsis-Induced Late-Phase Event of Acute Kidney Injury: A Narrative Review. *Pharmaceuticals* (2022) 15:899.

41. Zuo H, Cattani-Cavalieri I, Musheshe N, Nikolaev VO, Schmidt M. Phosphodiesterases as therapeutic targets for respiratory diseases. *Pharmacol \& Ther* (2019) 197:225–242.

42. Engoren M, Jewell ES, Douville N, Moser S, Maile MD, Bauer ME. Genetic variants associated with sepsis. *PLoS One* (2022) 17:e0265052.

43. Youle RJ, Narendra DP. Mechanisms of mitophagy. *Nat Rev Mol cell Biol* (2011) 12:9–14.

44. Glauser L, Sonnay S, Stafa K, Moore DJ. Parkin promotes the ubiquitination and degradation of the mitochondrial fusion factor mitofusin 1. *J Neurochem* (2011) 118:636–645.

45. Müller-Rischart AK, Pilsl A, Beaudette P, Patra M, Hadian K, Funke M, Peis R, Deinlein A, Schweimer C, Kuhn P-H, et al. The E3 ligase parkin maintains mitochondrial integrity by increasing linear ubiquitination of NEMO. *Mol Cell* (2013) 49:908–921.

46. Yuan H, Perry CN, Huang C, Iwai-Kanai E, Carreira RS, Glembotski CC, Gottlieb RA. LPS-induced autophagy is mediated by oxidative signaling in cardiomyocytes and is associated with cytoprotection. *Am J Physiol Circ Physiol* (2009) 296:H470--H479.

47. Piquereau J, Godin R, Deschênes S, Bessi VL, Mofarrahi M, Hussain SNA, Burelle Y. Protective role of PARK2/Parkin in sepsis-induced cardiac contractile and mitochondrial dysfunction. *Autophagy* (2013) 9:1837–1851.

48. Mishra S, Knupp A, Szabo MP, Williams CA, Kinoshita C, Hailey DW, Wang Y, Andersen OM, Young JE. The Alzheimer’s gene SORL1 is a regulator of endosomal traffic and recycling in human neurons. *Cell Mol Life Sci* (2022) 79:162.

49. Karch CM, Goate AM. Alzheimer’s disease risk genes and mechanisms of disease pathogenesis. *Biol Psychiatry* (2015) 77:43–51.
